# Evidence of chromatin and transcriptional dynamics for cold development in peach flower bud

**DOI:** 10.1111/nph.18393

**Published:** 2022-08-10

**Authors:** Monica Canton, Cristian Forestan, Gianpiero Marconi, Esther Carrera, Claudio Bonghi, Serena Varotto

**Affiliations:** ^1^ Department of Agronomy Food Natural Resources, Animals and Environment (DAFNAE) Agripolis University of Padova 35020 Legnaro PD Italy; ^2^ Department of Agricultural and Food Sciences (DISTAL) University of Bologna 40127 Bologna BO Italy; ^3^ Department Agricultural, Food and Environmental Sciences University of Perugia 06121 Perugia Italy; ^4^ Instituto de Biologıa Molecular y Celular de Plantas (IBMCP) Universidad Politecnica de Valencia‐Consejo Superior de Investigaciones Cientıficas (CSIC) Valencia Spain

**Keywords:** bud dormancy, ChIP‐Seq, DNA methylation, *Prunus persica*, RNA‐Seq

## Abstract

- In temperate zones, fruit trees regulate their annual growth cycle to seasonal environmental changes. During the cold season, growth is limited by both environmental and genetic factors. After the exposure to low temperature and fulfillment of chilling requirements, mild temperatures promote the growth and flowering. However, an insufficient chilling exposure may lead to nonuniform blooming, with a negative impact on fruit set.
- To gain insights into flower development in the fruit tree buds, peach is an interesting model, the flower and vegetative bud being distinct organs. To understand how flower bud development is regulated, we integrated cytological observations and epigenetic and chromatin genome‐wide data with transcriptional changes to identify the main regulatory factors involved in flower development during chilling accumulation.
- We demonstrated that growth cessation does not occur in peach flower buds during chilling accumulation, but that there are changes in transcript abundance of key genes of hormone metabolism and flower bud development, distribution of histone modifications (H3K4me3 and H3K27me3) and DNA methylation.
- Altogether, our findings indicate that during the cold season the flower bud is in a nondormant state and that the chilling experience allows flower differentiation to be completed.

## Introduction

Our current understanding of the cellular and molecular mechanisms involved in the seasonal regulation of bud growth in woody trees in temperate regions is based mainly on vegetative bud studies (Goeckeritz & Hollender, 2021). In many trees, short days and decreasing temperature during late summer/early autumn induce the cessation of bud growth. The most visible sign of vegetative bud growth cessation is the formation of an apical bud, consisting of the shoot apical meristem and leaf primordia enclosed by protective bud scales (Nitsch, 1957; Goffinet & Larson, 1981; Ruttink *et al*., 2007; Kayal *et al*., 2011). When in a dormant state, meristems and leaf primordia inside vegetative buds become insensitive to growth promotion signals, and growth arrest is maintained by endogenous signals; this stage is described as endodormancy. Toward the end of winter, in a further dormant state, called ecodormancy, growth arrest is primarily maintained by low temperatures (Lang *et al*., 1987; Anderson, 2015). Finally, after dormancy release, ‘warm’ temperatures promote bud burst. This terminology and progression of events have been indifferently applied in *Rosaceae* species having vegetative and flower buds (e.g. *Prunoidae* species) or mixed buds (e.g. *Pomoideae* species). However, Hatch & Walker (1969) and Walker (1970) suggested that peach flower buds have different resting mechanisms from those of vegetative buds. In peach, chilling is essential for the proper differentiation of the floral reproductive whorls. An insufficient chilling exposure may lead to abortion of the reproductive whorls, low bud burst and nonuniform blooming, with a negative impact on fruit set and quality (Luna *et al*., 1991; Atkinson *et al*., 2013). During the chilling period, cytological observations indicated that the reproductive whorls differentiate very slowly in the bud, but the major developmental events, including ovule formation in the carpel and microsporogenesis and pollen maturation in the anthers, occur at the end of the chilling period. The onset of microsporogenesis is therefore considered a good cytological indicator of chilling requirement (CR) fulfillment (Reinoso *et al*., 2002; Julian *et al*., 2011; Ríos *et al*., 2013). However, despite these anatomical observations, physiological and molecular investigations have been performed considering the peach flower bud as a dormant bud (see references in Goeckeritz & Hollender, 2021).

Bud dormancy is regulated by hormonal, transcriptional, epigenetic and physiological changes (reviewed in Singh *et al*., 2017). Abscisic acid (ABA) concentrations are highest during deep endodormancy and decrease by ecodormancy in response to chilling, with a trend that is opposite to that of gibberellins (GAs), whereas the role of cytokinin (CK) as a regulator of dormancy appears to be less documented. Until now, at the genetic level, a group of six tandemly repeated transcription factors of the MADS‐box gene family, named DORMANCY ASSOCIATED MADS‐box genes (DAM1–6), were identified in the peach genome as potential markers of the dormancy (Bielenberg *et al*., 2008). First, the DAM genes were identified in the *evergrowing* (*evg*) peach mutant. The *evg* trait is genetically heritable and segregates as a single recessive gene (Rodriguez *et al*., 1994). The *evg* locus was then identified and mapped in a genomic region of 132 kb that was demonstrated to be partially deleted in four of the six clustered MADS‐box genes. In e*vg*, DAM1–DAM4 were physically deleted, and the expression levels of DAM5 and DAM6 were reduced (Bielenberg *et al*., 2004, 2008), whereas in wild‐type peaches, DAM1, 4, 5 and 6 transcripts were downregulated in flower buds following dormancy release and differentially expressed in cultivars with different CRs (Leida *et al*., 2010, 2012). Low temperature was proposed to activate DAM transcription for dormancy induction by direct binding of the cold‐dependent C‐repeat binding factor (CBF) to DAM promoters (Saito *et al*., 2015; Niu *et al*., 2016; Zhao *et al*., 2018). The DAM genes were also identified in other species such as poplar (Ruttink *et al*., 2007), raspberry (Mazzitelli *et al*., 2007) and leafy spurge (Horvath *et al*., 2008). The six DAM genes of peach presumably originated from an ancestor related to the flowering transition regulator SHORT VEGETATIVE PHASE (SVP) of *Arabidopsis thaliana*, a transcriptional repressor that inhibits flowering by direct repression of the FLOWERING LOCUS T (FT) (Jiménez *et al*., 2009). In hybrid aspen, SVP has been characterized in vegetative buds as one of the major regulators of bud dormancy (Singh *et al*., 2018, 2019), but the functional role of SVP and DAM genes in *Rosaceae* remains to be elucidated.

The role of both epigenetic and chromatin regulation in DAM genes and bud dormancy has been studied in *Rosaceae* fruit trees. DNA methylation pattern variations were observed in sweet cherry flower buds in early winter (Rothkegel *et al*., 2020). In addition, it was observed that DAM genes are subject to chromatin regulation (Jimenéz *et al*., 2012; Leida *et al*., 2012; Niu *et al*., 2016). By considering possible commonalities between chromatin dynamics at the AtFLC locus and DAM gene loci during vernalization and winter bud dormancy, respectively, the distribution of chromatin marks, such as H3K4me3 and H3K27me3, were investigated in peach flower buds during endodormancy and ecodormancy by Zhu *et al*. (2020). They showed that expression of the five DAMs remains steadily unchanged with the ensuing warm temperature after chilling, and that this state of regulation correlates with robust increases of sRNA expression, H3K27me3 and CHH methylation, which is particularly pronounced in DAM4.

The regulation of both flower bud dormancy and bloom time in deciduous trees whose flower development spans the four seasons still requires more holistic studies. Understanding what regulates the physiological events occurring during flower bud dormancy and controls the fulfillment of CR is of particular importance in the context of global warming. Indeed, in recent years in milder regions, peach floral buds on trees do not always have their CR satisfied to complete development.

In this study, we focused our attention on flower bud development during winter in peach. To understand how bud development progression is regulated, we integrated cytological, epigenetic and chromatin genome‐wide data with transcriptional outputs to obtain a complete picture of the main regulatory pathways involved in flower development during chilling accumulation. To reach this goal we change our paradigmatic view of the flower bud status from dormant to nondormant during the cold season. Our findings support the hypothesis that in peach flower buds the chilling accumulation allows flower differentiation to be completed.

## Materials and Methods

### Plant materials

Flower buds were collected at four time points during the autumn/winter from 10‐yr‐old trees of Fantasia (FAN) in the experimental farm ‘Lucio Toniolo’ of the University of Padova (45°21′04.8″N, 11°57′01.3″E). FAN is a nectarine cultivar with medium/high winter CR (*c*. 770 chilling units (CU)) (Linsley‐Noakes & Allan, 1994). Samples were collected on 5 November 2018, 23 November 2018, 10 December 2018 and 7 January 2019 in autumn/winter 2018–2019, corresponding to 0, 200, 475 and 770 CU, respectively, calculated as described by Richardson *et al*. (1975) (Supporting Information Fig. S1). Daily maximum and minimum temperatures were retrieved from ARPAV (https://www.arpa.veneto.it/). At each time point, buds were collected from three groups each composed of three to four independent plants of the same genotype, corresponding to three biological replicates. During sampling, bud scales were removed from the buds, which were immediately frozen in liquid nitrogen and stored at −80°C until subsequent molecular analyses.

### Cytological observation and *in situ* hybridization

Buds from the time points 0, 475 and 770 CU were fixed in 4% paraformaldehyde (Sigma) in 0.1 M phosphate buffer (pH 7.2) for 16 h at 4°C and embedded in Paraplast Plus (Sigma‐Aldrich). Sections (7–10 μm) were cut using a microtome (RM2135; Leica Microsystems, Heidelberg, Germany) and collected on xylene‐coated slides (Superfrost Plus™ Adhesion Microscope Slides; Thermo Fisher Scientific, Gerhard Menzel BV & Co. KG, Braunschweig, Germany). For the cytological observation, slides were deparaffinized using xylene (Sigma‐Aldrich) and stained with 0.1% toluidine blue, and then air‐dried and mounted with DPX mounting medium (Honeywell Fluka, Charlotte, NC, USA). Callose deposition was observed using 0.1% of aniline blue. An *in situ* hybridization experiment was performed to localize the DAM4 expression domains and was conducted as previously described (Varotto *et al*., 2003). A detailed explanation of the protocol is reported in Methods S1 and Table S1.

### Hormone quantification

The concentrations of indol‐3‐acetic acid (IAA), ABA, GA (GA1 and GA4) and CKs (dihydrozeatin (DHZ), isopentenyl adenine (iP) and t‐zeatine (tZ)) were determined in dormant flower buds. The analysis was done in collaboration with the Servicio de Cuantificacion de Hormonas del IBMCP (Instituto de Biologia Molecular y Celular de Plantas) of the Universidad Politecnica de Valencia. A detailed explanation of all protocol steps is reported in Methods S1.

Statistical analysis was performed using R software. ANOVA was performed to identify the differences between the time points (cold effect), and then, when significant, Tukey's test with Bonferroni correction was applied. Differences were considered significant at the *P* ≤ 0.05 level.

### RNA sequencing (RNA‐Seq) and differentially expressed gene (DEG) identification

Transcriptome analyses were performed on total RNA isolated from flower buds at 0, 200, 475 and 770 CU; buds corresponding to replicates 2 and 3 were pooled, resulting in two biological replicates for each time point. Total RNA was extracted from 70–80 mg of frozen and ground flower buds using the RNeasy Plant Mini Kit (Qiagen) with minor modifications: 1.5% PVP‐40 was added to the extraction buffer RLT at a total volume of 750 μl instead of 450 μl. Indexed libraries for Illumina directional sequencing of total RNA were prepared using the TruSeq 3 Stranded RNA Library Prep Kit after rRNA depletion with Ribo‐Zero kit. Sequencing was performed at Novogene (HK) Co. Ltd (Hong Kong, China) on a NovaSeq 6000 platform. Details of sequencing and data analysis are reported in Methods S1 and Figs S2, S3 (Table S2).

### RNA‐Seq validation

Quantitative real‐time PCR expression analysis was performed on specific target genes such as *PpeDAM6*, *PpeDAM5*, *PpeDAM4*, *PpeDAM3*, *PpeDREB1D*, *PpeCYP707A4*, *PpeNCED5*, *PpeGA20ox* and *PpeUBQ*. A detailed explanation of the protocol is reported in Methods S1.

### Chromatin extraction and chromatin immunoprecipitation sequencing (ChIP‐Seq) analysis

Frozen flower buds at 0, 475 and 770 CU were finely powdered with liquid nitrogen and transferred to a 50 ml tube. Chromatin was extracted and immunoprecipitated according to Canton *et al*. (2022).

A total of 15 ChIP libraries (two biological replicates × two antibodies (Abs) for the sample 0 and 770 CU and three biological replicates × two Abs for the sample at 475 CU) and one control library (input) representing whole chromatin (WC) were used for the ChIP‐Seq assay. Library preparation and sequencing were performed by IGA Technology Services (Udine, Italy). Immunoprecipitated DNA was quantified using a Qubit 2.0 Fluorometer (Invitrogen) and an Ovation^®^ Ultralow V2 DNA‐Seq Library Preparation Kit (NuGEN, Redwood City, CA, USA) was used for library preparation following the manufacturer's instructions. Final libraries were checked with both the Qubit 2.0 Fluorometer and Agilent Bioanalyzer DNA assay or Caliper (PerkinElmer, Waltham, MA, USA). Libraries were then prepared for sequencing and sequenced on NovaSeq6000 (Illumina, San Diego, CA, USA), producing 30–70 M of 75 bp single‐end reads. After sequencing and filtering/trimming steps, high‐quality reads were mapped to the *Prunus persica* genome v.2.0 (Verde *et al*., 2017). ChIP‐Seq enrichment was calculated using a model‐based analysis of ChIP‐Seq (MACS; Feng *et al*., 2012). For each histone modification, we calculated the enriched peaks in each single time point, in order to have a genome‐wide view. More details are reported in Methods S1 and Fig. S4.

### DNA extraction, library preparation and sequencing for DNA methylation analysis

The DNA was isolated from three biological replicates of flower buds, sampled at four time points (0, 200, 475 and 770 CU) using the DNeasy^®^ Plant Mini Kit (Qiagen). The DNA libraries were prepared for each biological replicate (Table S3) and the protocol was performed according to Marconi *et al*. (2019). A total of 36 libraries were produced by double restriction–ligations, each using *Mse*I in combination with one of the three methylation‐sensitive enzymes *AciI*, *PstI* and *EcoT22I*, for the CG, CHG and CHH (H = A, C or T) contexts, respectively (Table S4). For each library, 45 M paired‐end reads of 150 nucleotides were generated (Table S5). Raw reads from the Illumina sequencing of the CG, CHG and CHH libraries were analyzed following the genome‐dependent pipeline (Marconi *et al*., 2019) and the sequences were mapped to the *P. persica* genome v.2.0 (Verde *et al*., 2017) obtained from Ensembl (http://plants.ensembl.org/index.html). Differentially methylated positions (DMPs) were therefore identified as sites that showed significant differences in the degrees of methylation between the time points (200 vs 0, 475 vs 0 and 770 vs 0), using logistic regression following the methylkit manual best practices package in R. Genomic regions with coregulated methylation changes upon chilling accumulation were identified by an adjacent dynamic window approach that targeted adjacent (at least two) significant DMPs (FDR, ≤ 0.05) with concordant methylation changes. After validation by logistic regression, the identified genomic regions were investigated as differentially methylated regions (DMRs; Marconi *et al*., 2019). Statistical analyses were performed in R v.3.3.2 (www.r‐project.org) using the psych, stats and gplots packages. A gene ontology (GO) enrichment analysis was performed on the DMGs using gProfiler (https://biit.cs.ut.ee/gprofiler/gost). Details of sequencing and data analysis are reported in Methods S1.

## Results

### Chilling accumulation period is marked by microspore mother cell development

**Fig. 1:**
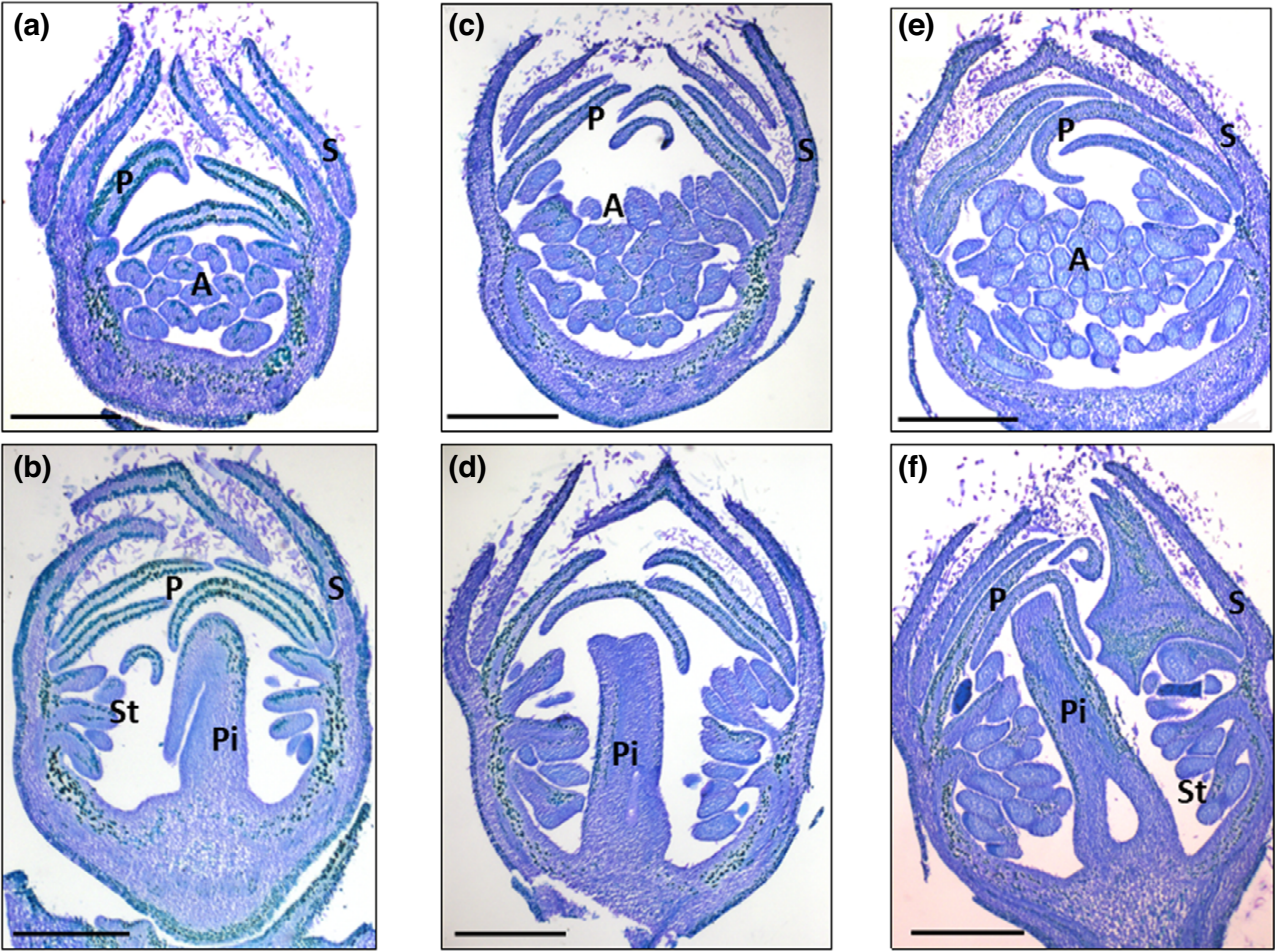
Longitudinal sections of peach buds during flower development. Images represent floral buds at 0 (a, b), 475 (c, d) and 770 chilling units (CU) (e, f) in median (b, d, f) and longitudinal anther (a, c, e) sections. Buds were stained with 0.1% toluidine blue. A, anthers; P, petal; Pi, pistil; S, sepal; St, stamen. Bar, 500 μm.

Flower buds were sectioned and observed under a light microscope at 0, 475 and 770 CU in order to visualize the morphological modifications occurring during the chilling accumulation period (Fig. 1a–f). At 0 CU, flower buds had completely differentiated the four verticils. With chilling accumulation progression, buds increased their volume through cellular expansion and at 475 CU anther locules were visible, while stigma, style and ovary were recognizable in the pistil. By observing our bud section series, it was also evident that anthers do not arrest their development. At 770 CU when the CR is fulfilled in FAN (Linsley‐Noakes & Allan, 1994), but the external temperatures are still low and photoperiod still short, anthers contain the microspore mother cell (M) ready to undergo meiosis inside their locule. All these observations indicate that peach flower bud growth and development proceed slowly throughout chilling accumulation and no meiosis was observed inside the anther locule until 770 CU. Based on these observations, the period from 0 to 770 CU, traditionally defined by Lang *et al*. (1987) as ‘endodormancy’, was renamed ‘chilling accumulation’.

### Hormone quantification in flower buds

**Fig. 2:**
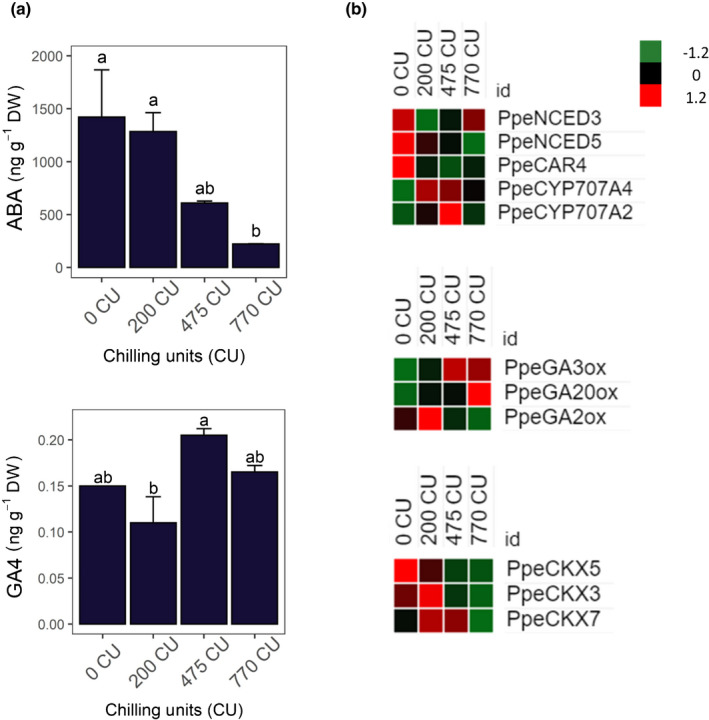
(a) Quantification of abscisic acid (ABA) and gibberellin 4 (GA4) during chilling accumulation in peach floral buds. Error bars indicate standard deviation (±SD) and letters indicate differences between the media value at each time point. Differences were considered significant at *P* ≤ 0.05 calculated using Tukey's test. (b) Heat maps of gene expression. Genes responsible for ABA and GA biosynthesis and degradation, and cytokinin (CK) degradation. Heat maps show the *z*‐score scaled gene expression levels (fragments per kilobase of transcript per million mapped reads) for the four samples.

The quantity of IAA, ABA, GA1, GA4, DHZ, iP, and tZ was determined in FAN flower buds at 0, 200, 475 and 700 CU (Table S6). Significant differences were found between time points in ABA and GA4 (Fig. 2a). In FAN flower buds, ABA appeared to decrease rapidly during chilling accumulation, while the GA4 increased mainly at 475 CU. No significant differences were observed for GA1, IAA, DHZ, iP and tZ (Fig. S5).

### Differentially expressed genes in response to chilling accumulation

To investigate transcriptional changes occurring in the peach flower bud during chilling accumulation, we performed a transcriptomic‐wide analysis using the RNA‐Seq approach at 0, 200, 475, and 770 CU. High‐quality reads were mapped on the *Prunus persica* v.2.0 reference genome (Table S7) and used for transcriptome re‐annotation (reference annotation‐based transcript, RABT). Into a two‐dimensional space, the principal component analysis (PCA) revealed that the first two PCs explain most of the variance (87%), and samples from each time point are projected together, indicating the high quality of the biological replicates (Fig. S6a). The new RABT allowed the identification of 30 342 loci corresponding to 50 399 transcripts. The re‐annotation identified 9154 splice variants, 1818 new intergenic transcripts and 314 antisense transcripts mapping with opposite orientations with respect to reference transcripts (Fig. S6b).

**Fig. 3:**
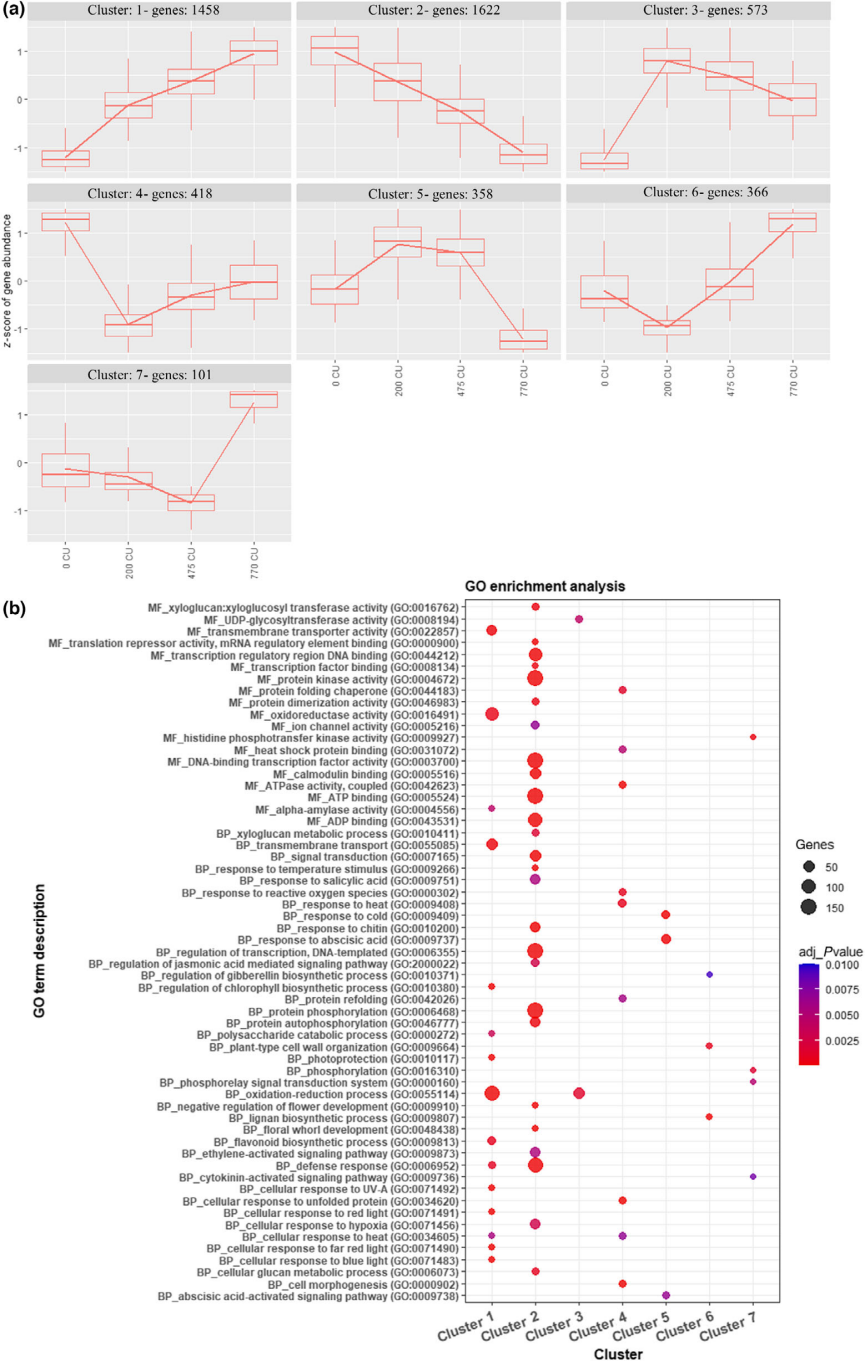
(a) Hierarchical clustering of expression dynamics for differentially expressed genes (DEGs) in peach floral buds. Expression values were normalized, and *z*‐scores of DEGs in each cluster are reported in the boxplots. (b) Gene ontology (GO) enrichment analysis separated by molecular function (MF) and biological process (BP) in all DEGs divided by clusters. We considered only GO terms with *P* < 0.01.

Subsequently, by using DESeq2 and a threshold of 0.01 on the adjusted *P*‐value and log_2_fold‐change > ¦0.585¦ (corresponding to 1.5 fold‐change variations in expression level), we identified 4896 DEGs during the time course of chilling accumulation (Dataset S1). We further investigated whether specific genes or signaling pathways could be associated with the different chilling accumulation during winter. To do this, we performed a hierarchical clustering of the 4896 DEGs in peach flower buds based on their expression in all samples. The DEGs were grouped into seven different clusters, with 1458 genes, 162 genes, 573 genes, 418 genes, 358 genes, 366 genes and 101 genes, respectively (Fig. 3a). To explore the functions and pathways associated with the gene clusters, we performed a GO enrichment analysis using gProfile for each of the seven identified clusters in flower bud (Fig. 3b; Dataset S2). Several GO terms were enriched, including response to temperature stimulus (GO:0009266, cluster 2), response to cold (GO:0009409 clusters 1 and 5), response to ABA (GO:0009737, cluster 5) floral whorl development (GO:0048438, clusters 2 and 5) and regulation of GA biosynthetic process (GO:0010371, cluster 6).

The DEGs related to hormone synthesis, signaling and catabolism were identified. *PpeNCED3* and *PpeNCED5*, responsible for ABA biosynthesis, showed higher expression between 0 and 200 CU. A similar expression profile was observed for *PpeCAR4* which encodes for an ABA receptor C2‐domain‐containing protein family. Finally, transcripts of two ABA hydroxylases (*PpeCYP707A2* and *PpeCYP707A4*), involved in the ABA catabolism, increased during chilling accumulation in flower buds (Fig. 2b; Dataset S3).

In flower buds, *PpeGA3ox* and *PpeGA20ox* genes are expressed from 475 to 770 CU. *PpeGA2ox*, responsible for GA catabolism, is expressed from 0 to 200 CU (Fig. 2b; Dataset S3). In addition to ABA and GA, three cytokinin oxidases (CKX3, CKX5 and CKX7), responsible for CK degradation, were found expressed during chilling accumulation (Fig. 2b; Dataset S3).

**Fig. 4:**
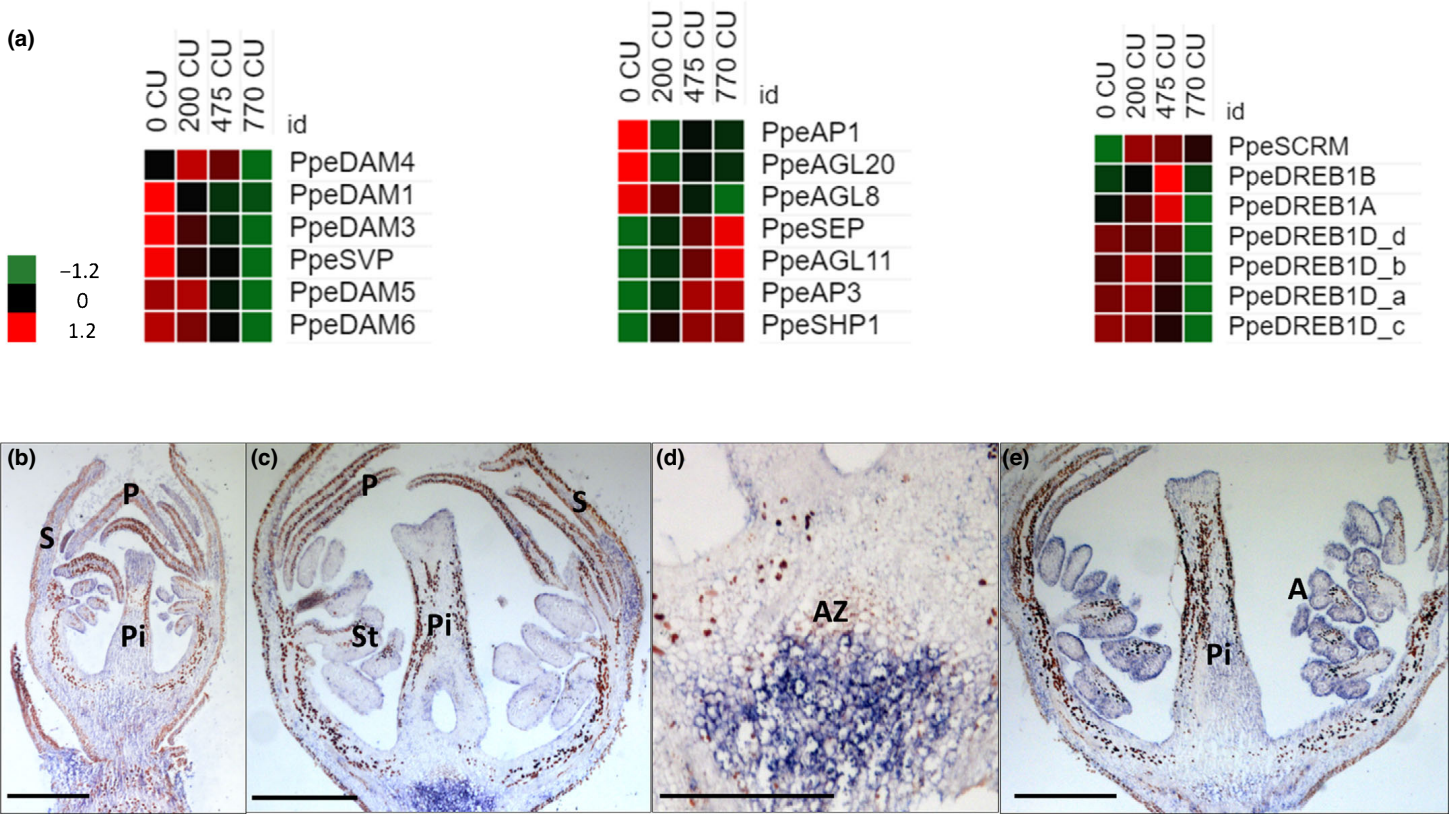
(a) Heat maps of DORMANCY ASSOCIATED MADS‐box genes (DAMs) and SHORT VEGETATIVE PHASE (SVP)‐like gene expression, floral MADS genes and genes involved in response to external stimuli in peach floral buds. Heat maps show the *z*‐score scaled gene expression levels (fragments per kilobase of transcript per million mapped reads) for the four samples. (b–e) *In situ* hybridization of *PpeDAM4* mRNAs in peach floral buds during chilling accumulation. Images represent longitudinal sections of a floral bud labeled with an antisense mRNA probe at 0 (b), 475 (c, d) and 770 chilling units (CU) (e). (d) A higher magnification of (c). The dark color represents the hybridization signal. A, anthers; AZ, abscission zone; P, petal; Pi, pistil; S, sepal; St, stamen. Bar, 500 μm.

All DAM genes are mainly expressed between 0 and 200 CU. Among the six annotated DAM genes, five were found to be differentially expressed: *PpeDAM1*, *PpeDAM3*, *PpeDAM5*, and *PpeDAM6* belong to cluster 2, and *PpeDAM4* is included in cluster 5 (Fig. 3a). In addition to DAM genes, an ortholog of Arabidopsis SVP was differentially expressed at the beginning of chilling accumulation and belongs to cluster 2, as do most DAM genes (Figs 3a, 4a; Dataset S3).

To confirm *PpeDAM6*, *PpeDAM5*, *PpeDAM4*, *PpeDAM3*, *PpeDREB1D*, *PpeCYP707A4*, *PpeNCED5* and *PpeGA20ox* gene expression, we performed a real‐time PCR (Fig. S7). All eight genes tested in RT‐qPCR showed a high correlation with the RNA‐Seq values. All four expressed DAM genes showed a very similar expression; they increase their expression until 200 CU and then decrease with the fulfillment of CR.

In addition to DAM and SVP genes, other putative MADS‐box coding genes were differentially expressed. In flowering plants, MADS‐box gene family members are key regulators of flowering time and floral organ identity. Many MADS genes that are important for the specification of flower whorls, as stated by the ABCDE model (Wells *et al*., 2015), were differentially expressed during chilling accumulation (Canton *et al*., 2021) All DEGs belonging to the A class were clustered in cluster 2, which means that their expression decreases during chilling accumulation. Conversely, all B‐, C‐ and E‐class genes were grouped in cluster 1 and therefore their expression increases during winter (Fig. 3a; Table S8). Genes belonging to the D class were not found to be expressed.

The Apetala 1 (AP1) (Irish & Sussex, 1990) belongs to the A‐class homeotic function and, together with two Agamous‐like gene transcripts, AGL8 and AGL20, rapidly decrease their expression after 0 CU. Conversely, the transcript abundance of Apetala 3 (AP3) (Jack *et al*., 1992), which encodes a class B function, playing a crucial role in petal and stamen development, together with AGL11 and Shatterproof 1 (SHP1) (Colombo *et al*., 2010), which are members of class C, and Sepallata (SEP) increased during the fulfillment of CR (Fig. 4a; Dataset S3).

Cold acclimation is pivotal to guarantee flower organ development. Many genes belonging to cold‐response pathways are differentially expressed at the end of the chilling accumulation period. Four different CBF/DREB1D (C‐repeat‐binding factor/dehydration responsive element‐binding factor 1D) transcripts are upregulated in the initial phase and progressively decrease their expression towards chilling accumulation. We also identified a CBF/DREB1A and a CBF/DREB1B with a similar expression pattern. In addition, the expression of ICE1, also known as SCREAM (SCRM1) (Chinnusamy *et al*., 2003), increases during the chilling accumulation period (Fig. 4a; Dataset S3).

Based on its different expression trend from the other DAM genes and its function in peach (Zhu *et al*., 2020), *DAM4* was chosen for *in situ* hybridization analysis. Longitudinal sections of flower buds were hybridized with *DAM4* antisense and sense probes (Figs 4b–e, S8, respectively). The *DAM4* transcript was present at all three time points analyzed (0, 475 and 770 CU). At 0 CU, DAM4 was ubiquitously expressed in flower tissues. At 475 CU the signal was detectable in all flower tissues; however, a stronger hybridization signal was visible in the abscission zone cells (AZ; Julian *et al*., 2007). With the progression of chilling accumulation (from 475 to 700 CU) the transcript was more detectable in pistil and anther epidermal tissues.

### Global changes in H3K4me3 and H3K27me3 during chilling accumulation

**Fig. 5:**
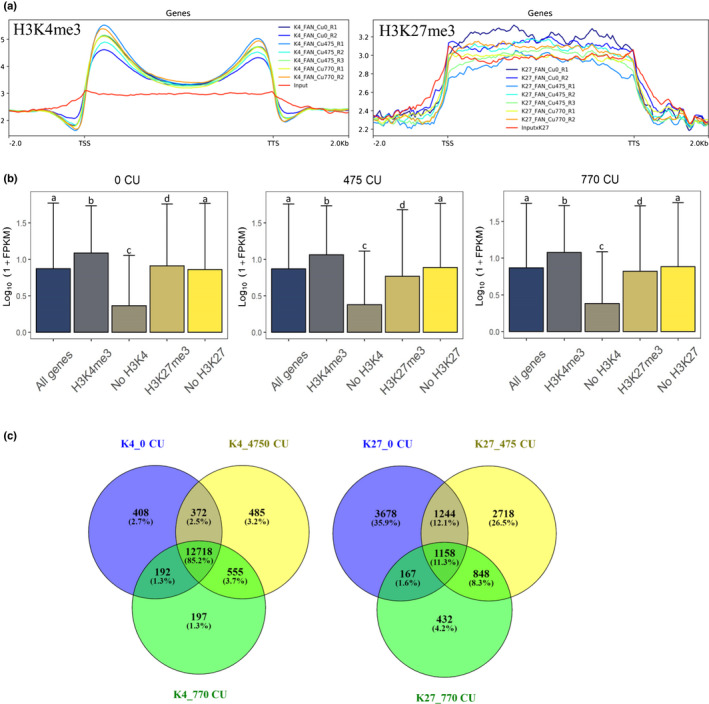
Summary of chromatin immunoprecipitation sequencing (ChIP‐Seq) analysis and integration of histone mark enrichment and gene expression results in flower peach bud. (a) Metagene plots showing the peak distribution of H3K4me3 and H3K27me3 along the transcribed genic regions extended by 2 kb at both ends in peach. TSS, transcription start site; TTS, transcription termination site. (b) Histogram plots report the median group expression and bars represent the interquartile range together with the median value. In each analyzed sample, the average expression levels of the genes marked by each histone modification are compared with the expression of the whole peach gene set. Letters indicate differences between media values at each time point determined by Tukey's test. FPKM, fragments per kilobase of exon per million reads mapped. Heat map showing expression levels and H3K4me3 and H3K27me3 abundance of all peach genes during cold accumulation series. Genes are grouped into five bins based on their expression levels, and the median histone mark abundance for each bin is plotted. Normalized histone modification levels for each annotated gene in each sample were defined as reads per million of reads (RPM) for H3K4me3 and reads per million per kilobase (RPKM) for H3K27me3. (c) Venn diagram of shared genes between all three analyzed time points.

To understand the chromatin mark (H3K4me3 and H3K27me3) dynamic on peach flower buds during the chilling accumulation, ChIP‐Seq was performed at 0, 475 and 770 CU. As expected, H3K4me3 was preferentially located at the transcription start site (TSS), whereas H3K27me3 was found throughout the entire gene (Fig. 5a). A peak in distribution for H3K4me3 was observed also close to the transcription termination site (TTS), probably because of a short distance between the genes (the median length of the intergenic region between two adjacent genes is < 2000 bp). To better understand the genome‐wide abundance of the two histone marks, we selected the peaks from −100 to 1000 bp from TSS for H3K4me3 and in the gene body for H3K27me3 (Datasets [Link], [Link]) and the associated gene in every single condition. We observed that the number of genes with at least one peak remained basically constant during chilling accumulation, with the major number of genes having at least one H3K4me3 peak in comparison to those enriched for the H3K27me3 histone mark (Table 1).

**Table 1:** Number of genes in H3K4me3 and H3K27me3 in each single condition in peach floral buds.

	H3K4me3	H3K27me3
0 CU	13 690	6247
475 CU	14 130	5968
770 CU	13 662	7360

The gene number was selected from −100 to 1000 bp from the transcription start site for H3K4me3 and in the gene body for H3K27me3.

By plotting the gene expression level of genes with or without associated H3K4me3 and H3K27me3 peaks, we observed that both histone modifications correlate globally with gene expression. At all three time points, genes with a H3K4me3 peak were more highly expressed than those without the peak in this histone mark, confirming the H3K4me3 positive correlation with gene expression. In addition, genes with a H3K27me3 peak were less highly expressed at both 475 and 770 CU time points, whereas at 0 CU the H3K27me3 correlation with gene expression was less evident (Fig. 5b; Dataset S6).

This was supported by analysis of histone mark abundance in relation to gene expression (Fig. 5c): an increase in gene expression correlates with a progressive increase of H3K4me3, whereas a clear trend is not detectable for H3K27me3, probably as a result of the low average level of this mark in our samples. Using a Venn diagram, we were able to identify the genes with a presence of H3K4me3 and H3K27me3 peaks that were shared between the time points; 85.2% of genes with a H3K4me3 peak maintain at least one peak at all three time points, whereas only 11.3% of the genes were marked by H3K27me3 at all three time points (Fig. 5d).

To investigate the impact of cold on chromatin marks, differential peak calling was performed in pairwise comparisons to identify genes with a statistically significant change in histone modification enrichment. Identified genes associated with differentially enriched peaks were therefore intersected with DEGs, identified in the RNA‐Seq analysis and functionally annotated based on the *Arabidopsis thaliana* homolog. The largest number of genes with a differential H3K4me3 peak associated with DEGs was observed at 770 CU in the comparison 0 vs 770 CU. On the other hand, the highest number of genes with a change in H3K27me3 was detected at 0 CU in the 0 vs 475 CU comparison (Table 2).

**Table 2:** Number of genes with at least one differential enriched peak of H3K4me3 and H3K27me3 associated with differentially expressed genes (DEGs) in each pairwise comparison in peach floral buds.

Comparisons	Enriched at:	H3K4me3	H3K27me3	Hypergeometric test, H3K4me3	Hypergeometric test, H3K27me3
0 vs 475 CU	0 CU	32	195	*P* < 7.128e−15	*P* < 2.005e−11
	475 CU	251	23		
475 vs 770 CU	475 CU	65	52	*P* < 7.768e−10	*P* < 2.299e−15
	770 CU	12	221		
0 vs 770 CU	770 CU	365	89	*P* < 5.169e−28	*P* < 4.909e−15
	0 CU	106	184		

**Fig. 6:**
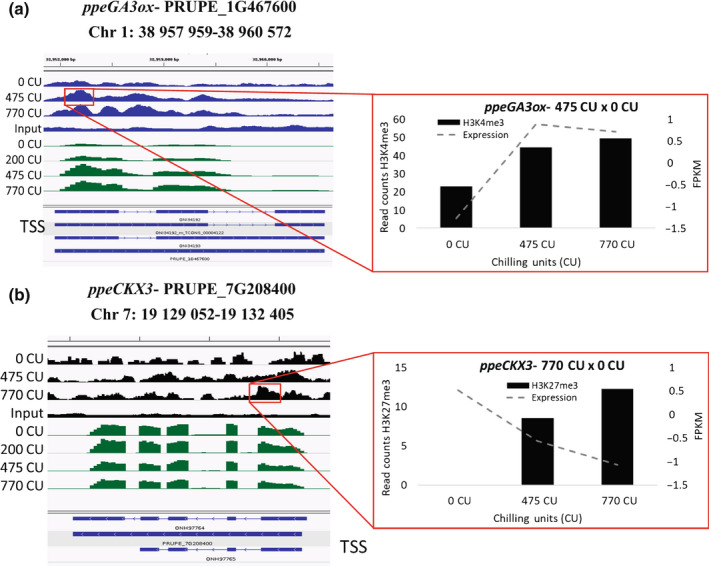
(a, b) Joined technical replicates were loaded into the Integrative Genomics Viewer (IGV) genome browser to visualize both H3K4me3 (a) and H3K27me3 (b) peaks along the gene together with gene expression (RNA‐Seq) peaks in the peach genome. Green peaks represent the RNA‐Seq distribution, blue peaks represent H3K4me3 and black peaks represent the H3K27me3 distribution along the gene (blue rectangles). TSS, transcription start site.

Our analysis identified a set of genes with a H3K4me3 differentially enriched peak related to hormone signaling, such as *PpeGA3ox* and *PpeGA20ox* (Fig. 6a; Dataset S7). Both genes had an increase in H3K4me3 and in gene expression at 475 vs 0 CU, indicating that this histone modification is associated with the upregulation of these genes (Santos‐Rosa *et al*., 2002; Howe *et al*., 2017). For the *PpeGA3ox* gene, another two H3K4me3 peaks were identified as differentially enriched at 770 vs 475 CU (Dataset S7). We also identified two CKX genes with a peak variation of H3K27me3. CKX genes are responsible for cytokinin degradation. The first one is a *PpeCKX3* in which we can observe an increase of H3K27me3 during chilling accumulation and a concomitant decrease in its expression level (770 vs 0 CU; Fig. 6b), suggesting that the H3K27me3 presence could be responsible for silencing the *PpeCKX3* transcription. The second gene is a CKX5, whose expression decreases during chilling accumulation together with the histone mark (Dataset S7). The *PpeNCED5* has an increase of H3K27me3 at 770 CU compared with 475 and 0 CU and a concomitant decrease of gene expression. *PpeDAM4* and *PpeDAM5* were found to have H3K4me3 peak variation. Both genes show a significant decrease of H3K4me3 at 770 CU compared with 475 CU (*PpeDAM4*) and 0 CU (*PpeDAM5*; Dataset S7).

We also observed changes in the histone modification in a gene that plays an important role in flowering time regulation and flower morphology establishment. A floral homeotic gene, Apetala 3 (AP3), was found to be enriched in H3K27me3 (770CU vs 475CU; Dataset S7), even if this differential enrichment was associated with an increase in its expression at 770 CU.

Another group of cold‐induced genes includes genes involved in callose synthase. Two genes were found with H3K4me3 peaks, Callose synthase 2 (*PpeCALS2*) and Callose synthase 11 (*PpeCALS11*). *PpeCALS2* has a H3K4me3 peak at 475 vs 0 CU and then gene expression decreases (Dataset S7). In turn, for *PpeCALS11*, a H3K4me3 peak was found at 770 vs 0 CU in which both histone mark and gene expression increase with the chilling accumulation (Dataset S7).

### Methylation changes during chilling accumulation

**Fig. 7:**
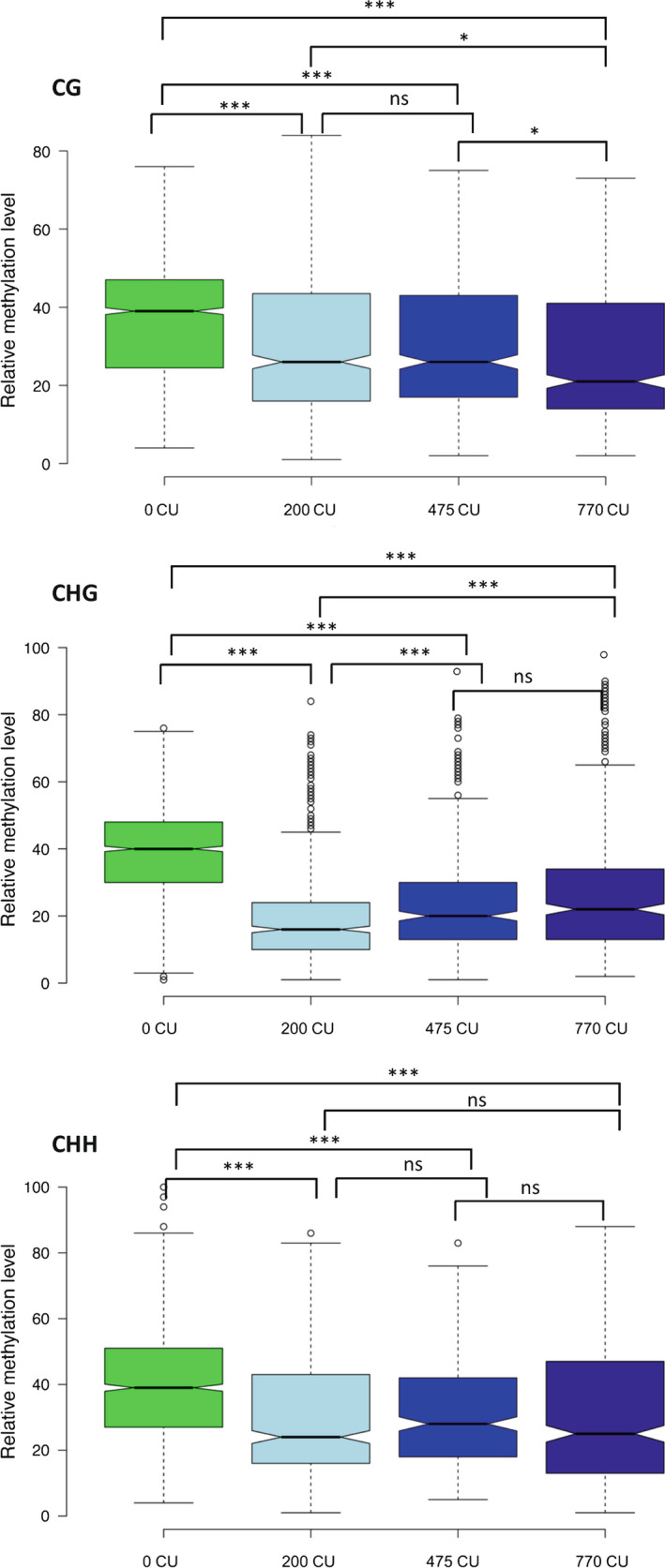
Box plot of relative degree of methylation during chilling accumulation in CG, CHG and CHH methylation contexts in peach floral buds. Asterisks indicate significance: *, *P* < 0.05;  ***, *P* < 0.0001; ns, nonsignificant, calculated using the *t*‐test. In the box plots, the boundary of the box closest to zero indicates the 25^th^ percentile, a black line within the box marks the median, and the boundary of the box farthest from zero indicates the 75^th^ percentile. Whiskers above and below the box indicate the 10^th^ and 90^th^ percentiles, respectively. Points above and below the whiskers indicate outliers outside the 10^th^ and 90^th^ percentiles, respectively.

The methylation content‐sensitive enzyme double digest restriction site‐associated DNA (MCSeEd) technique (Marconi *et al*., 2019) was used to identify DNA methylation changes during the chilling accumulation period. A relative level of methylation was calculated to identify the DMR distribution throughout chilling accumulation. PCA of the DMRs in CG, CHG and CHH at 200, 475 and 770 CU vs 0 CU revealed the high quality of the biological replicates (Fig. S9). Differences in methylation were observed at 200, 475 and 770 CU vs 0 CU in all three methylation contexts, CG, CHG and CHH, in which a decrease in relative methylation was observed from 0 CU (Fig. 7).

Enrichment analysis was performed using the binomial distribution of MCSeEd loci as expected and DMRs as observed. The DMRs were counted in pairs (200 vs 0 CU, 475 vs 0 CU and 770 vs 0 CU) according to their position in different genomic regions, such as the intergenic region, 2.5 kb windows upstream of TSS in which the gene promoter is located, within the gene body, and 2.5 kb windows downstream of the TTS. Significant differences were observed mainly in the gene body and in the intergenic region where they were preferentially mapped (Fig. S10).

Moreover, we analyzed the DMR distribution by pairs (200 vs 0 CU, 475 vs 0 CU and 770 vs 0 CU) across the transcribed genic regions extended by 2.5 kb at both ends (EGBs). At CG sites the relative methylation distribution was maintained similar between all three comparisons, and only at 475 vs 0 CU at the beginning of TSS was there a methylation decrease at 0 CU and an increase at 475 CU (Fig. S11). Similar behavior was observed in CHG context, differing only at 770 vs 0 CU, in which the methylation decreased in the gene body at 770 CU (Fig. S11). In the CHH methylation context, the relative methylation was distributed in a very similar way across the transcribed region and decreased with CU accumulation (Fig. S11). The genes closest to transcribed genic regions extended by 2.5 kb at both ends (EGBs) were defined as DMGs. To dissect the role of methylation in the regulation of gene transcription, we considered only DMGs associated with the DEGs, identified by the RNA‐Seq approach. We found 3169, 2151 and 1450 DMGs in CG, CHG and CHH, respectively. When associated with the DEGs, the DMG number sin CG, CHG and CHH were reduced to 1146, 720, 438, respectively, corresponding to 36%, 33% and 30% of DMGs that were also differentially expressed. A GO analysis was performed for all three methylation contexts (Fig. S12). At the CG level we identified important GO terms like ‘regulation of abscisic acid biosynthetic process (GO:0010115)’ and ‘sepal giant cell differentiation (GO:0090392)’. A CHG level GO term such as ‘negative regulation of gene expression, epigenetic (GO:0045814)’ was identified and finally, but no less importantly, we identified at the CHH level the GO term ‘regulation of timing transition from vegetative to reproductive phase (GO:0048510)’ (Fig. S12; Dataset S8). To better understand the distribution of DMGs, we divided them into upstream TSS, gene body and downstream TTS. The highest number of DMGs associated with DEGs were found inside the gene body in comparison with the upstream and downstream genes in all three methylation contexts. In addition, the number of demethylated genes was higher than that of methylated genes (Dataset S9). The percentages of demethylated genes of the total DMGs associated with the DEGs were 66.49%, 79.30% and 65.98% at CG, CHG and CHH, respectively.

Given the fact that DNA methylation in the gene promoter (located upstream of TSS) plays a crucial role in gene expression (Bartels *et al*., 2018), we focused on DMGs with DMRs overlapping the TSS upstream region. As expected by the literature (Feng *et al*., 2010), several DMGs located within 2.5 kb upstream of the TSS have orthologs in other species. The gene *PpeCKX3* was identified as demethylated in the CHH context at 475 vs 0 CU. An Agamous‐like 19 (*PpeAGL19*) was found to be demethylated at 770 vs 0 CU in the CG methylation context. In Arabidopsis, this gene is responsible for the regulation of timing of the transition from the vegetative to the reproductive phase (Schönrock *et al*., 2006).

## Discussion

In temperate regions, annual peach flower bud development is strictly synchronized seasonally. At the end of summer, flower buds differentiate outer whorls (sepals and petals). In autumn, short days and low temperatures slow down flower inner whorl (stamina and carpel) differentiation, which proceeds during chilling accumulation. In late winter, male gametophyte formation precedes female gametophyte differentiation which occurs just before blooming (Reinoso *et al*., 2002). All our observations indicate that there is no growth cessation in peach flower buds during chilling accumulation. Even when some vegetative bud dormancy‐associated features in flower buds, such as high ABA : GA ratio (Fig. S13), and callose deposition (Fig. S14) are evident, flower organ differentiation proceeds inside buds. In flower buds, the ABA : GA ratio declines during winter (Fig. S13), as also reported by Hernández *et al*. (2021) in temperate regions. The initial high concentration of ABA is guaranteed by the expression of *PpeNCED3* and *PpeNCED5* at the beginning of chilling accumulation. In turn, GAs are degraded by *PpeGA2ox*, maintaining a high ABA : GA ratio. With the progression of chilling accumulation, ABA is hydrolyzed by *PpeCYP707A2* and *PpeCYP707A4*, while GA increase is correlated with the expression of *PpeGA3ox* and *PpeGA20ox*. All these observations confirm previous transcriptomic analyses on flower buds in *Prunus* species (Vimont *et al*., 2019; Yu *et al*., 2020; Canton *et al*., 2021). Based on our results, chilling accumulation also affects chromatin mark dynamics and contributes to hormone balance in peach flower buds; it promotes an increase in H3K4me3 in the chromatin of *PpeGA3ox*, a key enzyme in GA biosynthesis (Hedden & Phillips, 2000), which is associated with an increase in *PpeGA3ox* expression. No similar observation about *GA3ox* has been reported in the literature before. H3K27me3 was noted to be present in the *PpeNCED5* gene, a key enzyme involved in ABA biosynthesis (Iuchi *et al*., 2001; Martínez‐Andújar *et al*., 2011). During chilling accumulation, the transcription of *PpeNCED5* decreases with the increase of H3K27me3, suggesting that this histone mark might silence this gene transcription.

Vegetative bud dormancy is characterized by plasmodesmata closure, while resuming growth requires their opening by β‐1,3‐glucanases in many plant species (Tylewicz *et al*., 2018; Singh *et al*., 2019). In peach flower buds, some genes associated with callose synthesis were found to be upregulated. In particular, the transcription of *PpeCALS2* and *PpeCALS11* is associated with H3K4me3 during chilling accumulation. CASL11 has not been functionally characterized in peach until now; however, in Arabidopsis this gene product is responsible for callose formation to separate the micropores during meiosis in the anthers (Wu *et al*., 2018). For this gene, chromatin dynamics and expression patterns suggest a possible involvement in callose deposition around meiocytes in peach anthers, a deposition that, based on our results, occurs after chilling fulfillment.

Until now, the exact role of CALS2 is also unknown. However, by considering both the chromatin dynamics and expression pattern during chilling accumulation, we hypothesize that in peach buds its function might be correlated with callose synthesis to cease the communication between bud cells (Fig. S14), as demonstrated in hybrid poplar (Tylewicz *et al*., 2018). However, whereas in poplar, vegetative bud plasmodesmata closure is essential to growth arrest and dormancy, the functional meaning of callose deposition in peach flower buds needs further investigation as buds remain active during chilling accumulation. Other genes involved in plasmodesmata closure were found to be differentially expressed in our study (Dataset S10). The same genes were also expressed in hybrid aspen (Tylewicz *et al*., 2018).

The observation that flower buds continue their development during winter is confirmed by the dynamic expression patterns of MADS‐box genes responsible for the specification of inner flower whorls (Fig. 4a; Dataset S3). The continuous development of flower buds requires, first, a process of cold adaptation ensured by the transcript abundance increase of DREB1/CBFs, recognized as the master regulators for cold acclimation (Kidokoro *et al*., 2021). With the progression of chilling accumulation, the expression of genes associated with cold acclimation and ABA metabolism decreases, giving space to the upregulation of the floral homeotic genes SEP (class E) and Apetala 3 (AP3 class B) (Figs 2a, 4a). The expression of inner flower whorl identity genes allows the cold development of stamens and pistils, whereas gametophyte formation occurs only after chilling fulfillment as confirmed by the expression profile of genes involved in micro‐ and macrogametogenesis (Canton *et al*., 2021). In peach, bud dormancy (endo‐ and ecodormancy) traditionally includes all these development events that require active gene transcription, hormone metabolism and variation in epigenetic marks. Therefore, the Lang *et al*. (1987) definition of dormancy cannot be applied to peach flower bud. Following this consideration, what role can DAMs play, expression of which has been closely associated with the flower bud dormancy status (Zhu *et al*., 2020)? The DAM1 and 3‐5‐6 genes and a SVP‐like peach gene were upregulated at the onset of chilling accumulation, whereas their expression decreased during chilling accumulation. A different expression pattern was observed for DAM4, the transcript abundance of which, after a decrease at 200 CU, increases again until chilling fulfillment. These data are in agreement with Zhu *et al*.'s (2020) observations regarding peach DAM gene expression dynamics during winter and regarding the distinctive expression profile and possible function of DAM4 in peach flower bud. Interestingly, DAM4 transcript is localized in the flower bud AZs (Fig. 4b–e). Abscission zone cells are responsible for the dropping of freeze‐damaged buds, which can occur particularly after the fulfillment of CR (Julian *et al*., 2007). The role of DAM4 in the AZs remains to be elucidated, although its transcript abundance in AZ can be related to a mechanism of cold adaptation. So far, only fragmentary information is available regarding the relationship between DAM and cold adaptation, such as the interaction between DAM6 and CBF‐like factors in Japanese apricot (Zhao *et al*., 2018).

DORMANCY ASSOCIATED MADS‐box genes are known to be regulated by both H3K4me3 and H3K27me3 chromatin marks. In particular, this was observed for DAM1‐4‐6 with a decrease in H3K4me3 at the TSS and an increase of H3K27me3 in the gene body induced by chilling (Leida *et al*., 2012; Zhu *et al*., 2020). However, in our study we did not find any H3K27me3 enrichment in DAM gene chromatin during chilling accumulation. Similarly, for SVP in vegetative buds of hybrid aspen, researchers did not observe any significant increase of H3K27me3 marks at the SVP locus upon low temperature treatment (Singh *et al*., 2018). *PpeDAM4* and *PpeDAM5* were found to have H3K4me3 peak presence, the dynamics of which correlated well with their cognate expression. Indeed, H3K27me3 deposition may occur at later stages of flower development after CR fulfillment. This histone modification was found to be present in the chromatin of these genes, specifically in nondormant buds in sweet cherry and peach (Vimont *et al*., 2020; Zhu *et al*., 2020), while in our case flower buds were collected during chilling accumulation. It has recently been proposed that the mechanism for silencing DAM genes appears to be similar to the silencing mechanism of *AtFLC* during vernalization: repressive H3K27me3 marks at the DAM locus build up as chill accumulates (Goeckeritz & Hollender, 2021). However, based on our results the pattern of H3K4me3 accumulation and H3K27me3 depletion does not resemble that observed for the regulation of *AtFLC* during vernalization, owing to the absence of H3K27me3 deposition at DAM genes after cold exposure.

The role of histone modification H3K4me3 and H3K27me3 in chromatin regulation of genes in response to environmental and developmental cues in plants is well documented (Zhang *et al*., 2007, 2009; Rodier *et al*., 2011). Our study revealed that these two histone modifications are associated with chromatin dynamics during chilling accumulation, particularly of genes involved in ABA, GA and cytokinin metabolism, which is essential for correct progression of the flower developmental pattern. Additionally, H3K4me3 is associated with the expression of genes for callose deposition and DAM genes. The apparently lower correlation with the expression of specific genes observed for H3K27me3 might be a result of both its association with gene silencing and tissue specificity (Lloyd & Lister, 2021). Flower buds are composed of different tissues, and this can clearly affect the detection of chromatin marks on a gene having a tissue‐specific expression pattern.

In peach flower buds, the role of DNA methylation in chilling accumulation processes is less evident. A decrease of DNA methylation was observed in all sequence contexts during chilling accumulation. However, both GO terms and correlation between DRMs and gene expression indicated that DNA methylation might be involved in regulating hormone balance and expression of a few MADS genes in flower buds. In our study, the highest number of DMGs were found in the gene body, followed by upstream and downstream gene regions (Dataset S9). These results agree with Prudencio *et al*. (2018), who found most frequently mapped fragments within the gene coding regions in almond. In apple, global methylation and transcriptional analyses revealed that nonexpressed genes or genes expressed at low levels were highly methylated in the gene body regions, suggesting that gene body methylation is negatively correlated with gene expression (Xing *et al*., 2019). However, the function of gene body methylation in comparison to that occurring in the other genomic regions remains elusive (Bewick & Schmitz, 2017).

In conclusion, we produced a global picture of changes in transcriptome, H3K4me3, H3K27me3 and DNA methylation during chilling accumulation in peach flower bud. We uncover chromatin states that correlate with the transcript abundances of the key genes in hormone regulation and flower bud developmental progression, and concluded that during chilling accumulation flower bud differentiates the inner whorls of flowers so that after chilling fulfillment the male gametes can be produced before the end of winter. Further investigations are needed to clarify the difference in regulation of flower bud cold development and vegetative bud dormancy.

## Author contributions

CB and SV conceived this study. MC and CF collected plant material and conducted cytological analysis and preparatory experiments for RNA‐Seq, MCSeEd and ChIP‐seq. MC, CF and GM conducted the statistical analysis for RNA‐Seq, ChIP‐Seq and MCSeEd, respectively. EC performed the hormone quantification analysis. MC, CB and SV wrote the original draft. All authors contributed to manuscript writing and editing.

## Supporting information


**Dataset S1** FPKM values of RNA‐Seq analysis.

**Dataset S2** List of Gene Ontology (GO) terms enriched for the DEGs in each cluster.

**Dataset S3** Gene ID and expression values of selected genes.

**Dataset S4** List of peaks in each single condition and in pairwise comparisons for H3K4me3.

**Dataset S5** List of peaks in each single condition and in pairwise comparisons for H3K27me3.

**Dataset S6** Effect size calculation for H3K4me3 and H3K27me3.

**Dataset S7** List of selected genes with H3K4me3 and H3K27me3 peak presence.

**Dataset S8** List of GO terms for the differentially methylated genes.

**Dataset S9** Gene number of DMGs associated with DEGs.

**Dataset S10** Genes involved in plasmodesmata closure.

**Fig. S1** Flower bud development during the cold season.
**Fig. S2** Comparison between *PpDAM* loci gene annotation using the reference *Prunus persica* genome annotation and the reference annotation‐based transcript (RABT) annotation.
**Fig. S3** Prunus GO terms of reference and newly annotated transcripts were *de novo* annotated using Trinotate (Bryant *et al*., 2017) and compared with the prunus GO annotation available in the EnsemblPlants/Biomart database in July 2020 using the WEGO GO plotting tool categorized using level 2 of the GO lineage.
**Fig. S4** Correlation heatmaps between replicates using read count data were produced using diffbind package in R v.4.2.
**Fig. S5** Quantification of gibberellin 1 (GA1), indol‐3‐Acetic acid (IAA), isopentenyl adenine (iP), dihydrozeatin (DHZ) and t‐zeatine (tZ) during chilling accumulation.
**Fig. S6** Principal component analysis (PCA) of samples by transcriptome profile.
**Fig. S7** Gene expression validation in RT‐qPCR of *PpeDAM6*, *PpeDAM5*, *PpeDAM4*, *PpeDAM3*, *PpeDREB1D*, *PpeCYP707A4*, *PpeNCED5* and *PpeGA20ox*.
**Fig. S8** *In situ* hybridization of *PpeDAM4* mRNAs in peach floral buds during chilling accumulation.
**Fig. S9** Principal component analysis (PCA) of the differentially methylated regions (DMRs) in CG, CHG and CHH at 200 (ff9), 475 (ff11) and 770 CU (ff13) vs 0 CU (ff8).
**Fig. S10** Enrichment analysis of DMRs in different genomic regions.
**Fig. S11** DMR distribution by pairs (200 vs 0 CU, 475 vs 0 CU and 770 vs 0 CU) across the transcribed genic regions extended by 2.5 kb at both ends (EGBs) at the differentially methylated regions (CG, CHG, CHH contexts).
**Fig. S12** Gene ontology analysis (GO) of differentially methylated genes (DMGs) in all three different methylation contexts.
**Fig. S13** Histograms representing the ABA/GA4 ratio at the *P* ≤ 0.05 level at 0, 200, 475 and 770 chilling units (CU).
**Fig. S14** Longitudinal sections of peach buds during flower development.
**Methods S1** Methods, figures and tables.
**Table S1** List of primers employed in this work.
**Table S2** Prunus GO terms of reference and newly annotated transcripts were *de novo* annotated using Trinotate (Bryant *et al*., 2017) and compared with the prunus GO annotation available in the EnsemblPlants/Biomart database on July 2020 using the WEGO GO plotting tool categorized using level 2 of the GO lineage.
**Table S3** List of codes, index adaptors and oligonucleotides.
**Table S4** Characteristics of the restriction enzymes used for the MCSeEd technique.
**Table S5** Sequencing data summary of DNA methylation sequencing.
**Table S6** Means and standard deviations of hormone quantification.
**Table S7** RNA‐Seq summary statistics.
**Table S8** Genes belonging to the ABCDE model.
Please note: Wiley Blackwell are not responsible for the content or functionality of any Supporting Information supplied by the authors. Any queries (other than missing material) should be directed to the *New Phytologist* Central Office.

## Data Availability

The data that support the findings of this study are available in the Gene Expression Omnibus (accession no. GSE189882 for reference RNA‐Seq data (https://www.ncbi.nlm.nih.gov/geo/query/acc.cgi?acc=GSE189882) and accession no. GSE190586 for ChIP‐Seq data (https://www.ncbi.nlm.nih.gov/geo/query/acc.cgi?acc=GSE190586)). Data for DNA methylation sequencing (bioproject PRJNA787489) can be found in the National Library of Medicine (https://www.ncbi.nlm.nih.gov/bioproject/?term=PRJNA787489).
