# Supplementary material for: Evidence of chromatin and transcriptional dynamics for cold development in peach flower bud
Source: New Phytol. 2022 Aug 10;236(3):974–88. doi: 10.1111/nph.18393 (PMC9804738; doi:10.1111/nph.18393)
Supplement: Supplementary file 11 — Fig. S1 Flower bud development during the cold season. Fig. S2 Comparison between PpDAM loci gene annotation using the reference Prunus persica genome annotation and the reference annotation‐based transcript (RABT) annotation. Fig. S3 Prunus GO terms of reference and newly annotated transcripts were de novo annotated using Trinotate (Bryant et al., 2017) and compared with the prunus GO annotation available in the EnsemblPlants/Biomart database in July 2020 using the WEGO GO plotting tool categorized using level 2 of the GO lineage. Fig. S4 Correlation heatmaps between replicates using read count data were produced using diffbind package in R v.4.2. Fig. S5 Quantification of gibberellin 1 (GA1), indol‐3‐Acetic acid (IAA), isopentenyl adenine (iP), dihydrozeatin (DHZ) and t‐zeatine (tZ) during chilling accumulation. Fig. S6 Principal component analysis (PCA) of samples by transcriptome profile. Fig. S7 Gene expression validation in RT‐qPCR of PpeDAM6, PpeDAM5, PpeDAM4, PpeDAM3, PpeDREB1D, PpeCYP707A4, PpeNCED5 and PpeGA20ox. Fig. S8 In situ hybridization of PpeDAM4 mRNAs in peach floral buds during chilling accumulation. Fig. S9 Principal component analysis (PCA) of the differentially methylated regions (DMRs) in CG, CHG and CHH at 200 (ff9), 475 (ff11) and 770 CU (ff13) vs 0 CU (ff8). Fig. S10 Enrichment analysis of DMRs in different genomic regions. Fig. S11 DMR distribution by pairs (200 vs 0 CU, 475 vs 0 CU and 770 vs 0 CU) across the transcribed genic regions extended by 2.5 kb at both ends (EGBs) at the differentially methylated regions (CG, CHG, CHH contexts). Fig. S12 Gene ontology analysis (GO) of differentially methylated genes (DMGs) in all three different methylation contexts. Fig. S13 Histograms representing the ABA/GA4 ratio at the P ≤ 0.05 level at 0, 200, 475 and 770 chilling units (CU). Fig. S14 Longitudinal sections of peach buds during flower development. Methods S1 Methods, figures and tables. Table S1 List of primers employed in this work. Table S [file NPH-236-974-s001.pdf]

New Phytologist

***New Phytologist*** **Supporting Information**

Article title: **Evidence of chromatin and transcriptional dynamics for cold development in peach flower bud**

Authors: Monica Canton, Cristian Forestan, Gianpiero Marconi, Esther Carrera, Claudio Bonghi and Serena Varotto

Article acceptance date: 15 July 2022

The following Supporting Information is available for this article:

New Phytologist

**Fig. S1** Peach flower bud development during cold season. Images were photographed at 0 chilling units (CU), 200CU, 475CU, and 770CU from branches located at the median portion of the plant.

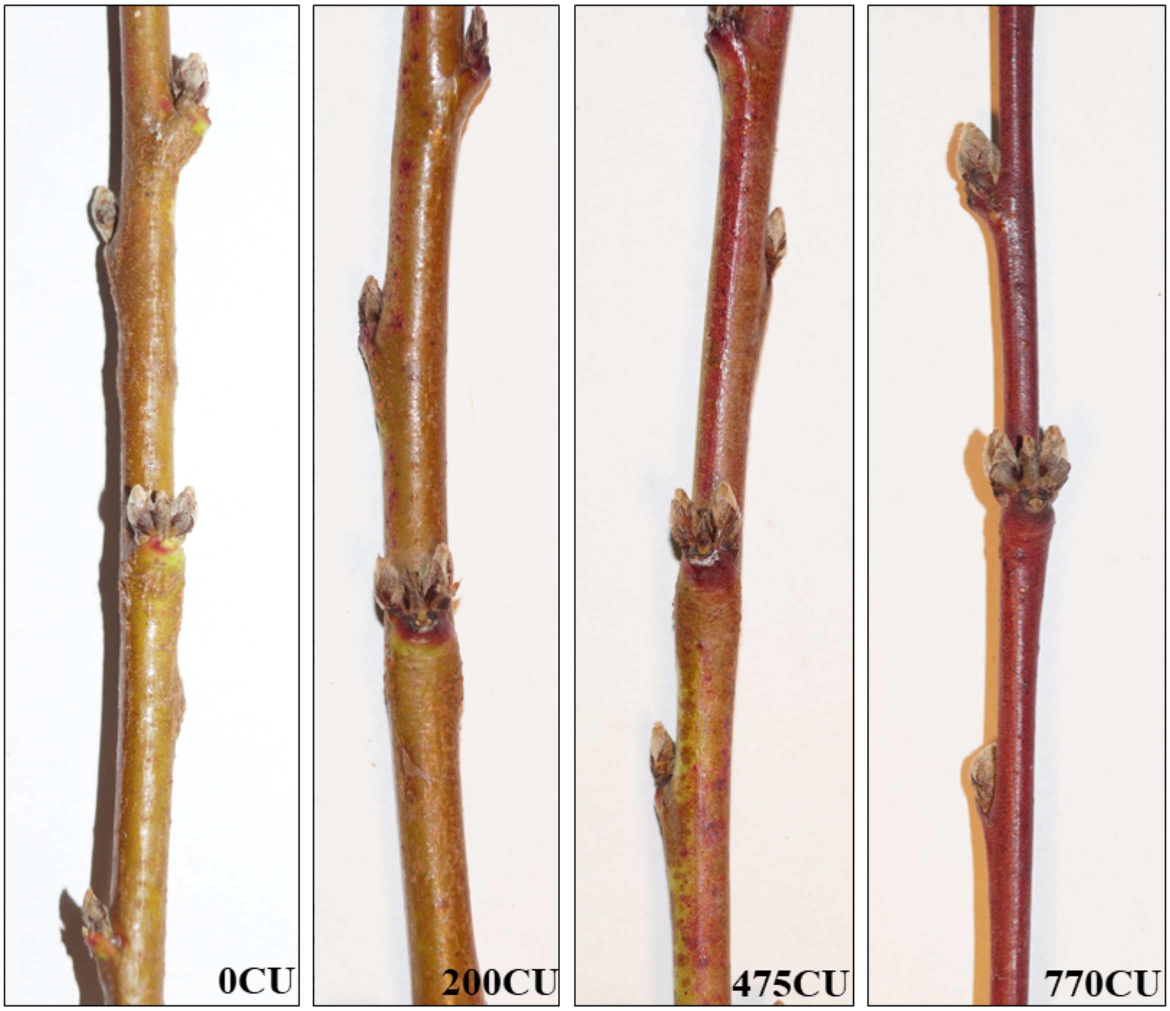

**Fig. S2** Comparison between *PpDAM* loci gene annotation using the refence *Prunus persica* genome annotation (blue rectangles) and Reference Annotation Based Transcript (RABT) annotation (red rectangles).

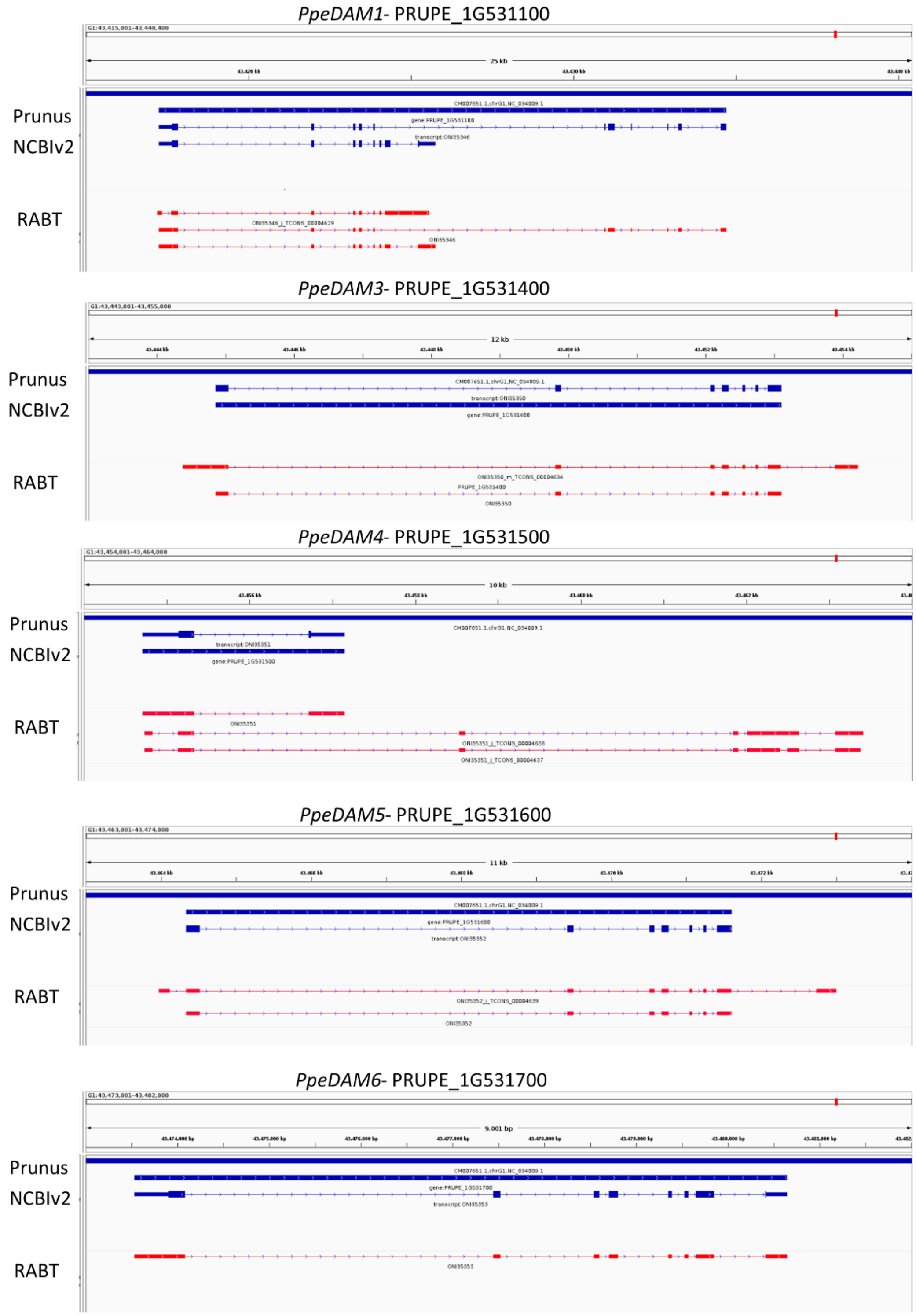

**Fig. S3** Prunus GO terms of reference and newly annotated transcripts were de-novo annotated using Trinotate (Bryant *et al*., 2017) and compared with the prunus GO annotation available at EnsemblPlants/Biomart database on July 2020 using WEGO GO plotting tool categorized using level 2 of the GO lineage.

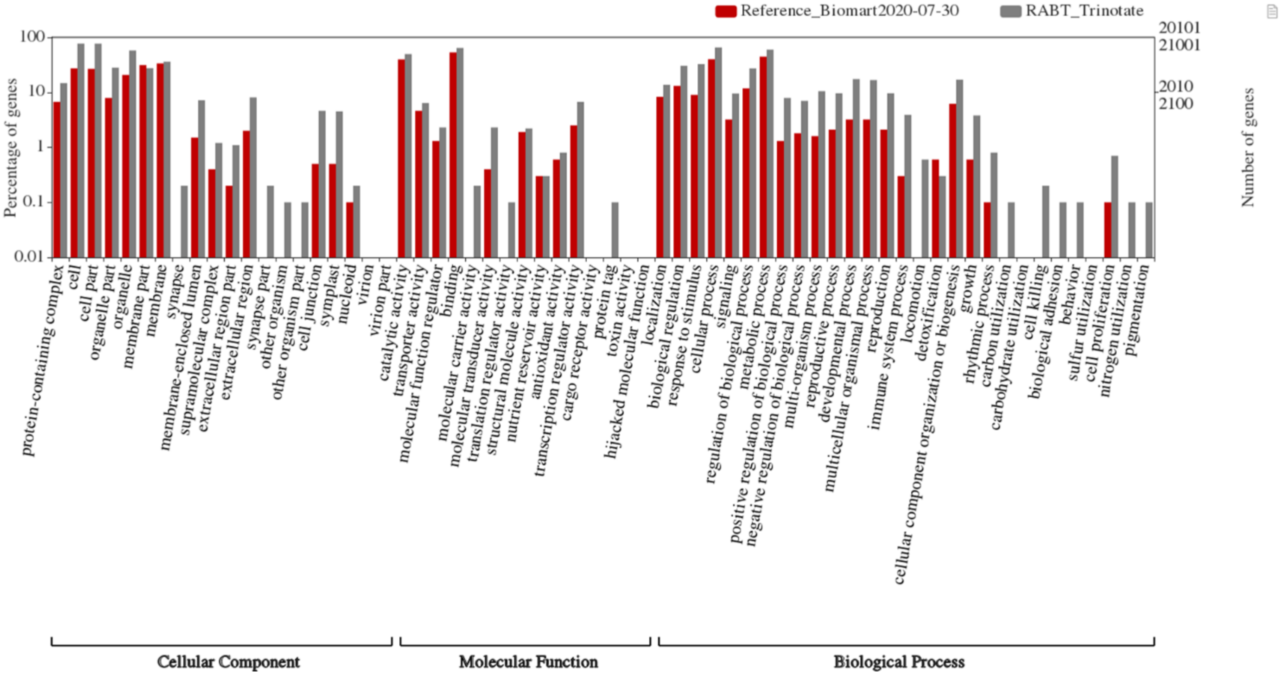

**Fig. S4** Correlation heatmaps between replicates, using read count data were produced using DiffBind R package version 4.2.

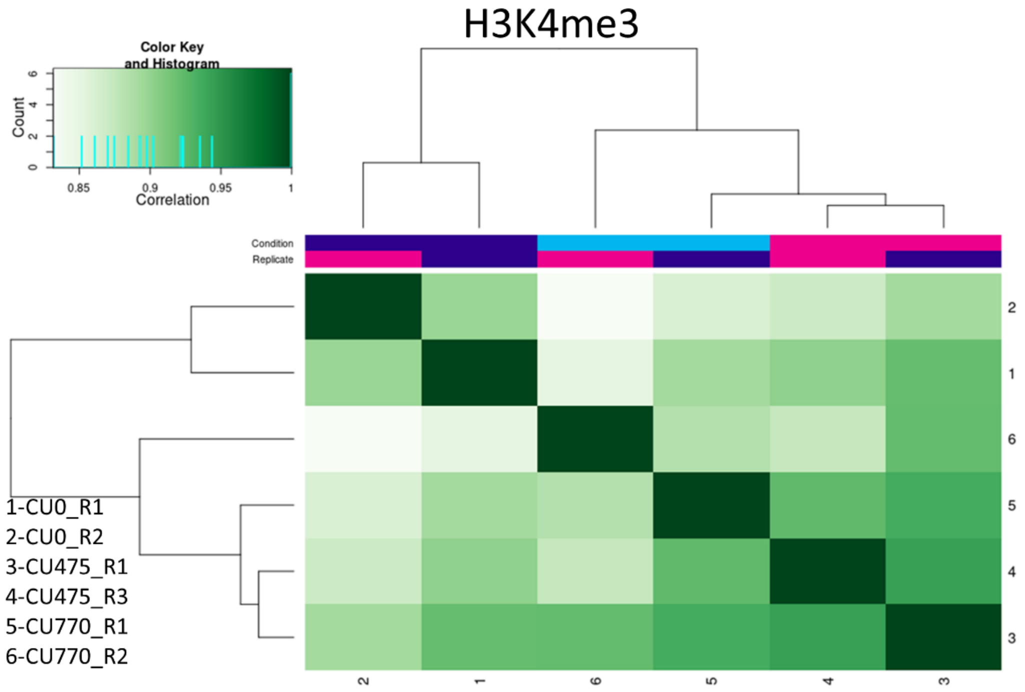


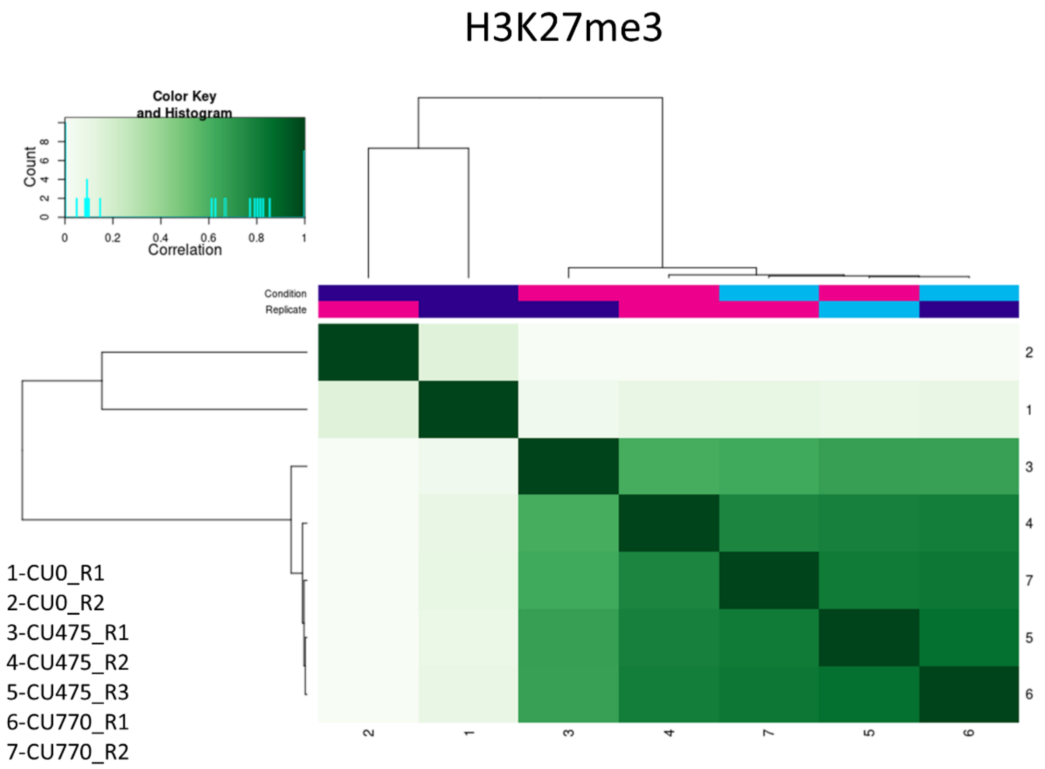

**Fig. S5** Quantification of Gibberellin 1 (GA1), Indol-3-Acetic acid (IAA), isopentenyl adenine (iP), dihydrozeatin (DHZ) and t-zeatine (tZ) during chilling accumulation. Error bars indicate standard deviation (+/- SD).

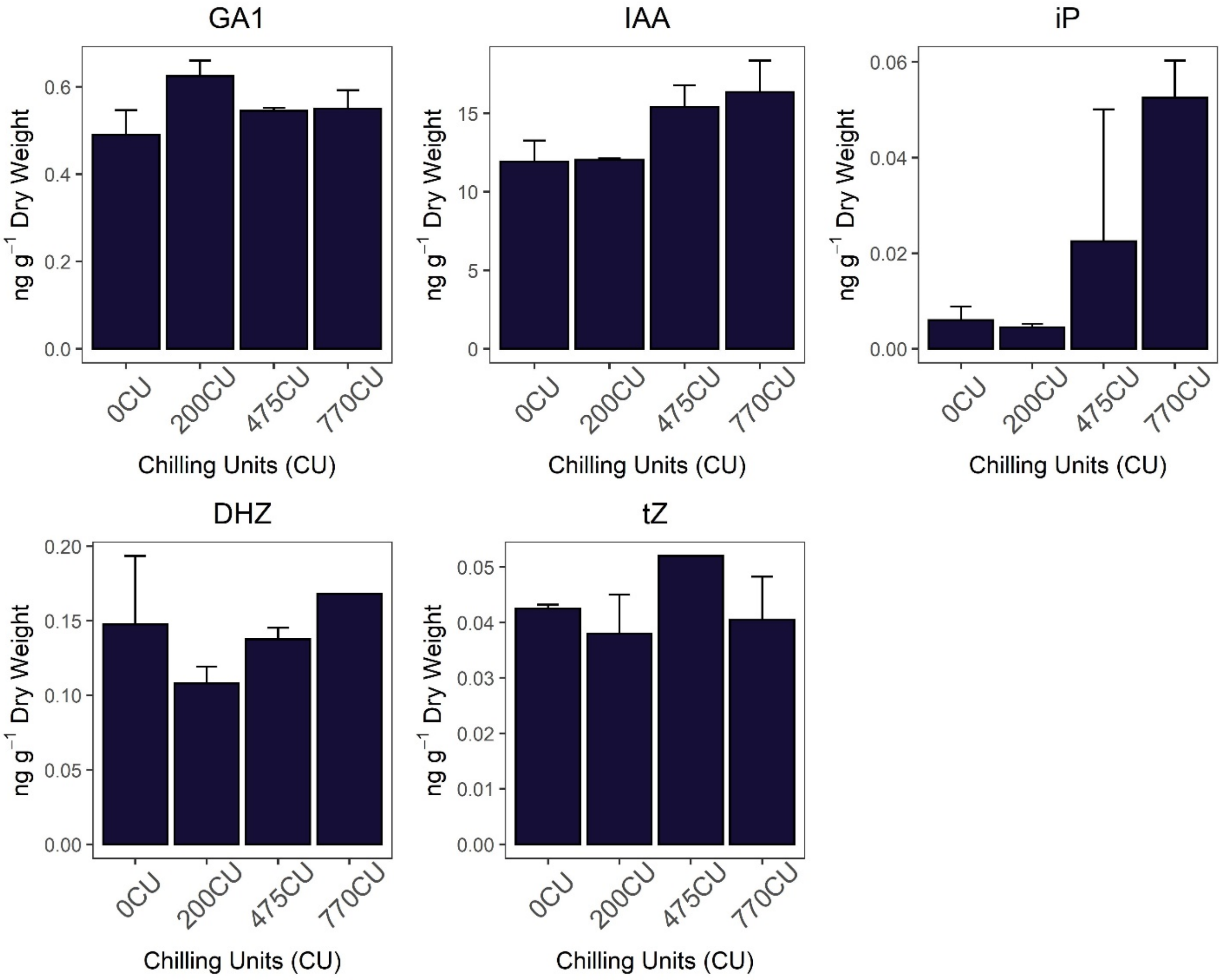

**Fig. S6** Principal component analysis (PCA) of samples by transcriptome profile. PC1 and PC2 represent the first two largest sample variances from overall gene expression (a). Identification and classification of novel genes using RABT approach (b).

(a)

(b)

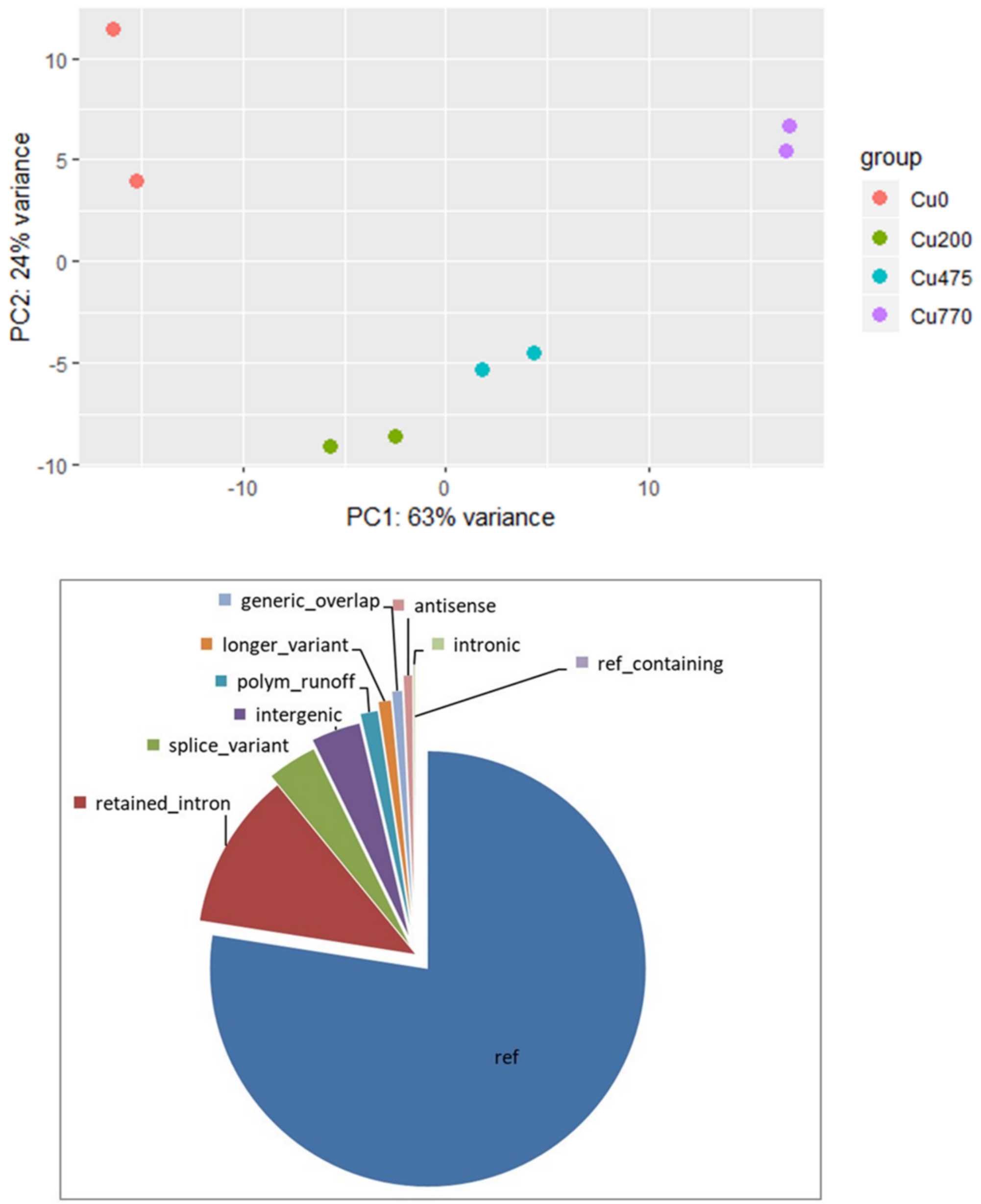

**Fig. S7** Gene expression validation in RT-qPCR of *PpeDAM6, PpeDAM5, PpeDAM4, PpeDAM3, PpeDREB1D, PpeCYP707A4, PpeNCED5* and *PpeGA20ox*. A correlation analysis was performed for each gene using the RNAseq-FPKM values and qPCR expression normalized to *PpeUBQ*. A Pearson correlation is reported for each gene. Error bars indicate standard error (+/- SE).

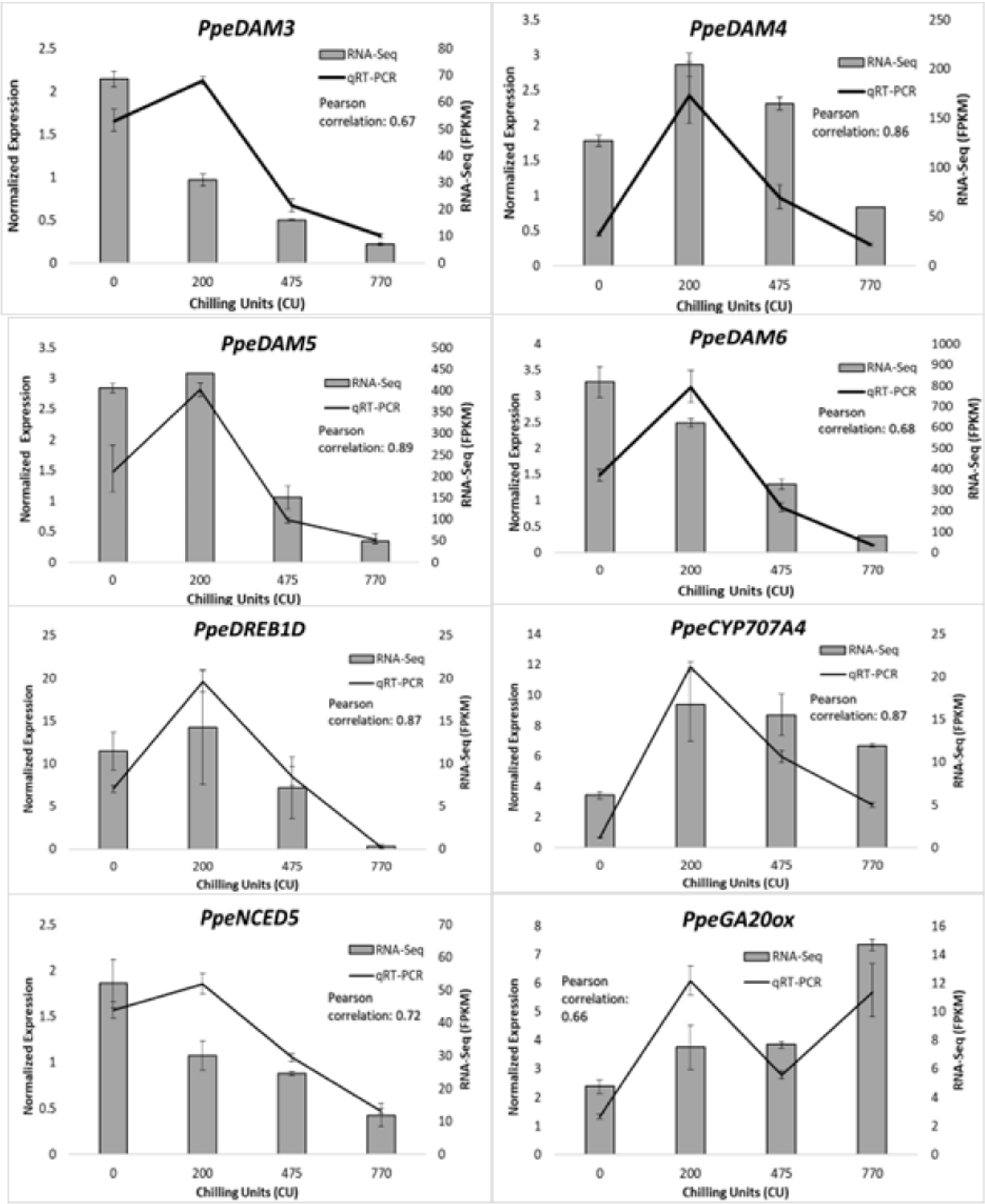

**Fig. S8** *In situ* hybridization of *PpeDAM4* mRNAs in peach floral buds during chilling accumulation. The image represents a longitudinal section of a floral bud labeled with a sense mRNA probe. Bars = 500μm.

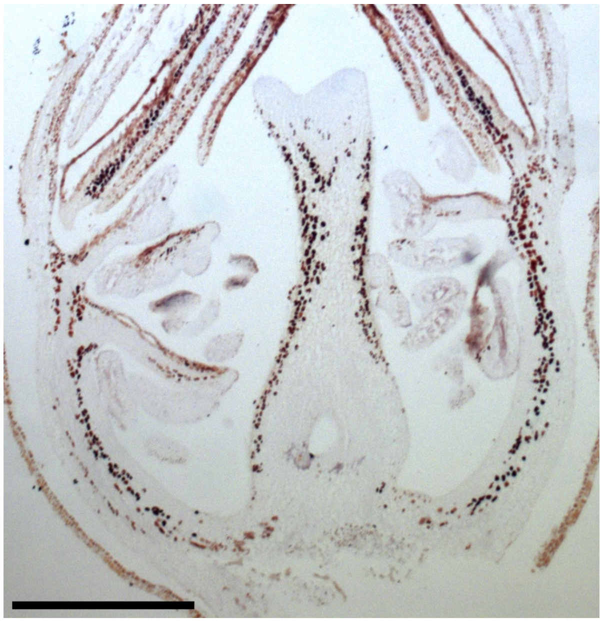

New Phytologist

**Fig. S9** Principal component analysis (PCA) of the differentially methylated regions (DMR) in CG, CHG and CHH at 200 (ff9), 475 (ff11) and 770CU (ff13) against 0CU (ff8). All three biological replicates are present.

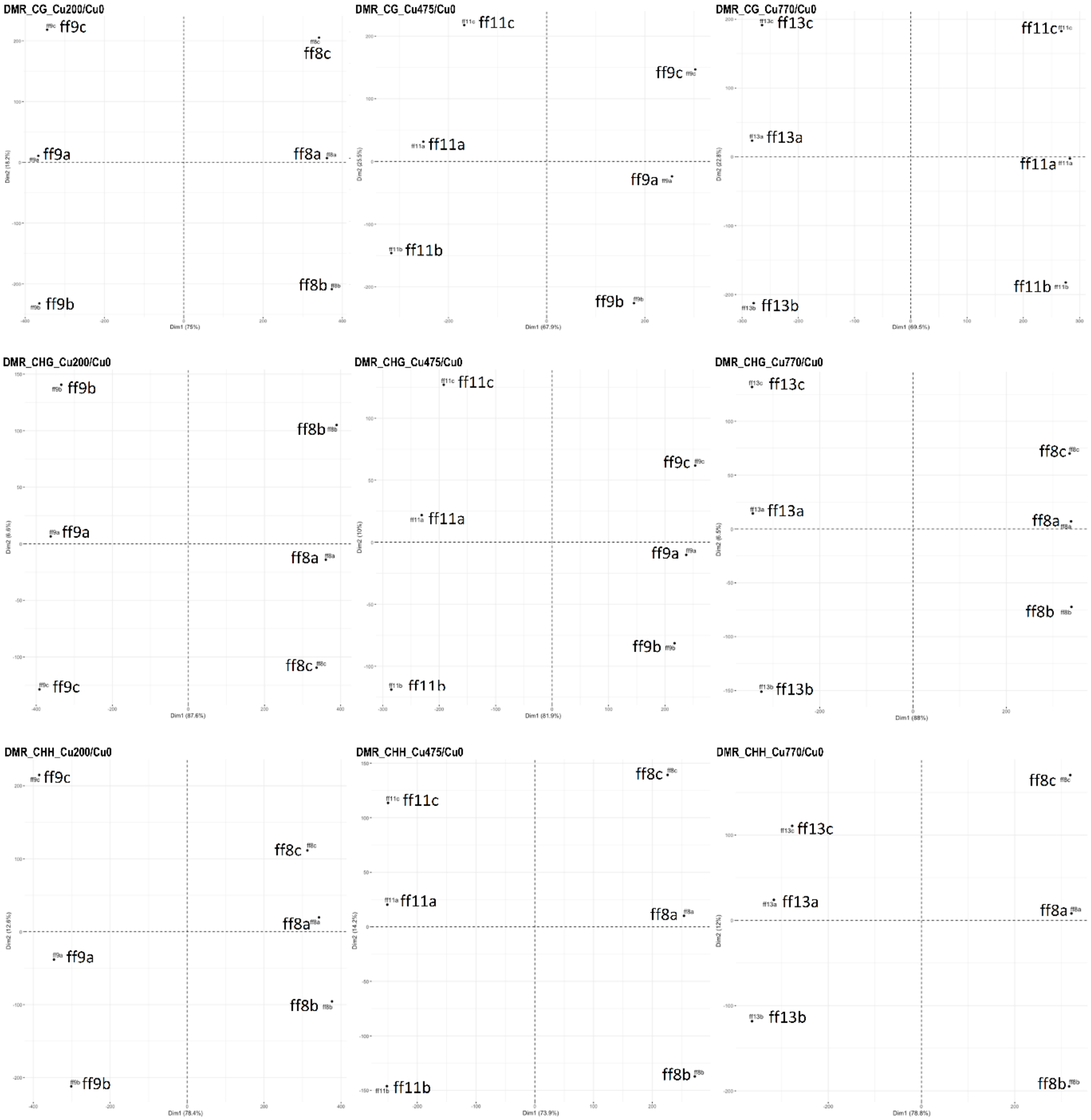

**Fig. S10** Enrichment analysis of DMRs in different genomic regions. Enrichment analysis was performed using the binomial distribution of all of the MCSeEd loci as expected and the differentially methylated regions (CG, CHG, CHH contexts; note that scales for each context differ), as the observed datasets. Light gray = expected number of DMRs, Dark gray = observed number of DMRs. Asterisks indicate significant (*p < 0.05), highly significant (**p < 0.001), and extremely significant differences (***p < 0.0001) and ns means non-significant, calculated using the t test.

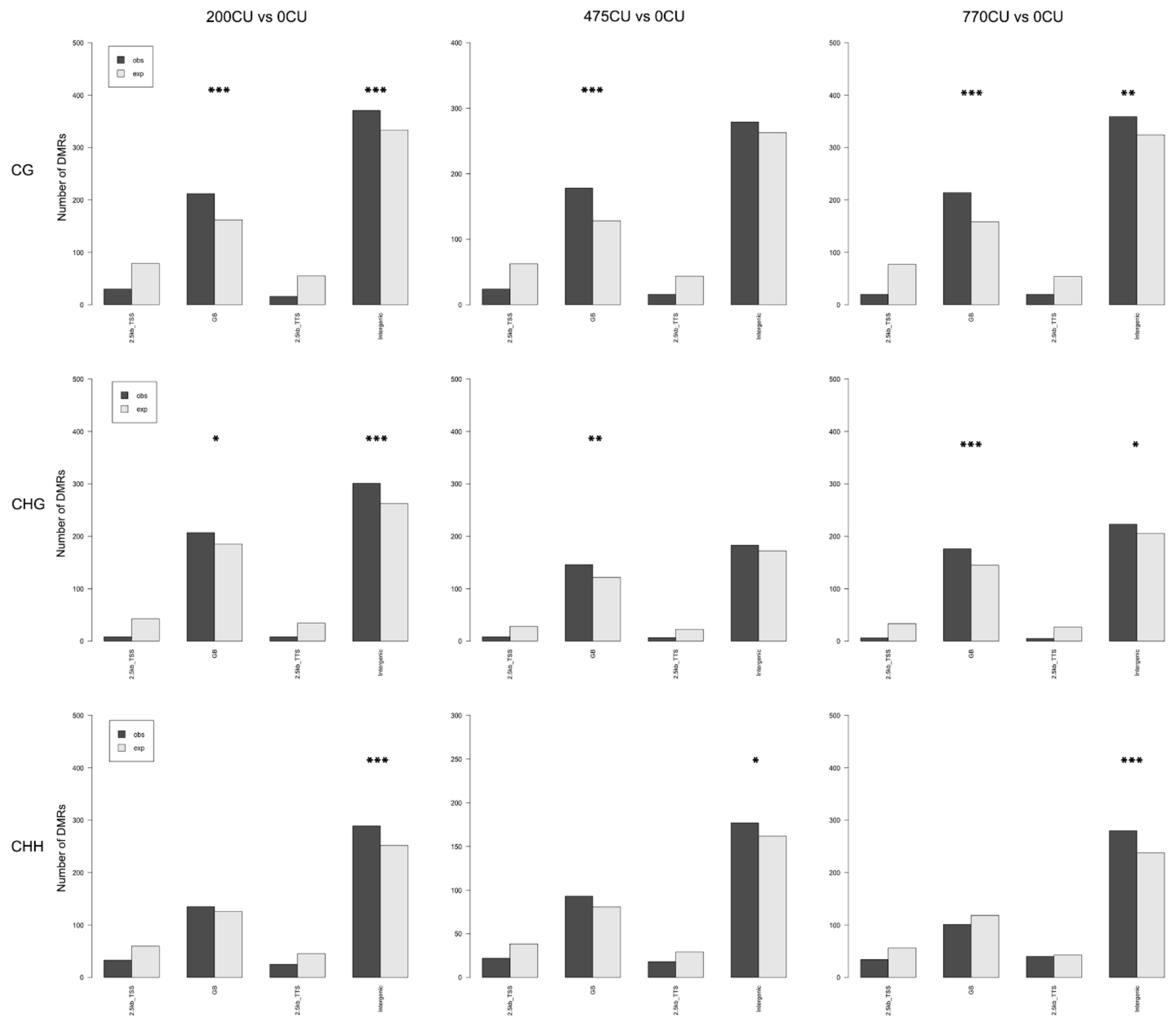

**Fig. S11** DMRs distribution by pairs (200CU *vs* 0CU, 475CU *vs* 0CU and 770CU *vs* 0CU) across the transcribed genic regions extended by 2.5 kb at both ends (EGBs) at the differentially methylated regions (CG, CHG, CHH contexts).

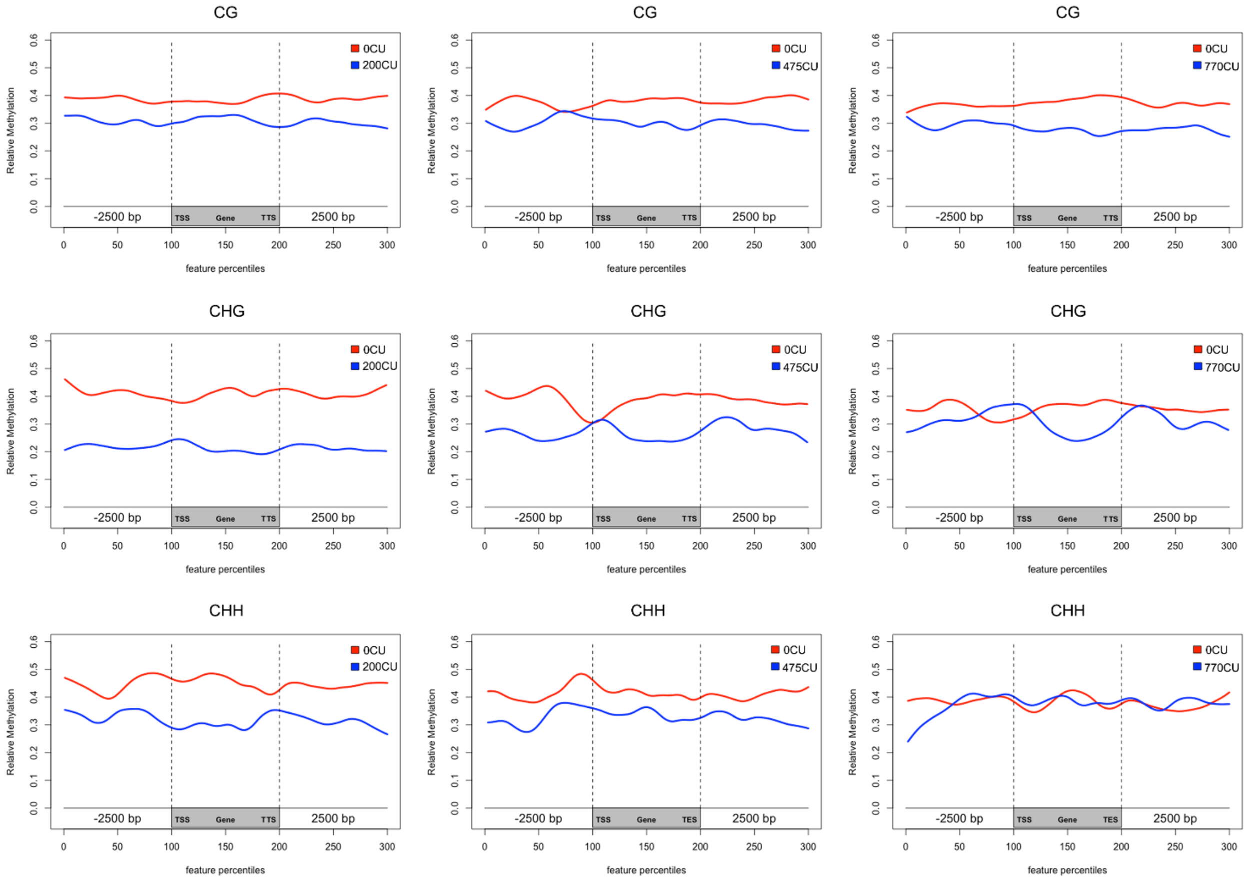

**Fig. S12** Gene Ontology analysis (GO) of Differentially methylated genes (DMG) in all three different methylation contexts.

**CG**

| ppersica.GO_BP.RABT11E10 | | 200CU x 0CU | 475CU x 0CU | 770CU x 0CU |
|---|---|---|---|---|
| Term Name | Term ID | p_adj | p_adj | p_adj |
| response to drug | GO:0042493 | 1.000 | $7.424\times10^{-3}$ | 1.000 |
| sepal giant cell differentiation | GO:0090392 | 1.000 | $1.913\times10^{-2}$ | 1.000 |
| plant epidermal cell fate specification | GO:0090628 | 1.000 | $1.913\times10^{-2}$ | 1.000 |
| self proteolysis | GO:0097264 | 1.000 | $2.074\times10^{-2}$ | 1.000 |
| regulation of abscisic acid biosynthetic process | GO:0010115 | $6.070\times10^{-2}$ | $3.003\times10^{-2}$ | 1.000 |
| response to water | GO:0009415 | $3.681\times10^{-2}$ | 1.000 | 1.000 |
| dipeptide transport | GO:0042938 | 1.000 | 1.000 | $4.119\times10^{-2}$ |
| negative regulation of seed germination | GO:0010187 | $2.584\times10^{-1}$ | 1.000 | $4.567\times10^{-2}$ |

1 to 8 of 8 Page 1 of 1

| ppersica.GO_CC.RABT11E10 | | 200CU x 0CU | 475CU x 0CU | 770CU x 0CU |
|---|---|---|---|---|
| Term Name | Term ID | p_adj | p_adj | p_adj |
| clathrin-coated pit | GO:0005905 | $4.121\times10^{-2}$ | $7.668\times10^{-2}$ | 1.000 |

1 to 1 of 1 Page 1 of 1

| ppersica.GO_MF.RABT11E10 | | 200CU x 0CU | 475CU x 0CU | 770CU x 0CU |
|---|---|---|---|---|
| Term Name | Term ID | p_adj | p_adj | p_adj |
| ubiquinol-cytochrome-c reductase activity | GO:0008121 | 1.000 | $1.109\times10^{-3}$ | $1.439\times10^{-1}$ |
| calcium-dependent cysteine-type endopeptidase activity | GO:0004198 | 1.000 | $1.160\times10^{-2}$ | 1.000 |
| tripeptide transmembrane transporter activity | GO:0042937 | $7.426\times10^{-1}$ | 1.000 | $1.923\times10^{-2}$ |
| peroxiredoxin activity | GO:0051920 | 1.000 | 1.000 | $2.694\times10^{-2}$ |
| mannitol transmembrane transporter activity | GO:0015575 | 1.000 | 1.000 | $3.295\times10^{-2}$ |
| myo-inositol transmembrane transporter activity | GO:0005365 | 1.000 | 1.000 | $3.295\times10^{-2}$ |
| D-ribose transmembrane transporter activity | GO:0015591 | 1.000 | 1.000 | $3.295\times10^{-2}$ |
| sorbitol transmembrane transporter activity | GO:0015576 | 1.000 | 1.000 | $3.295\times10^{-2}$ |
| D-xylose transmembrane transporter activity | GO:0015148 | 1.000 | 1.000 | $3.295\times10^{-2}$ |
| phospholipase A1 activity | GO:0008970 | $1.607\times10^{-1}$ | 1.000 | $3.979\times10^{-2}$ |
| oligopeptide transmembrane transporter activity | GO:0035673 | 1.000 | 1.000 | $4.274\times10^{-2}$ |

1 to 11 of 11 Page 1 of 1

**CHG**

| ppersica.GO_BP.RABT11E10 | | 200CU x 0CU | 475CU x 0CU | 770CU x 0CU |
|---|---|---|---|---|
| Term Name | Term ID | p_adj | p_adj | p_adj |
| negative regulation of anoikis | GO:2000811 | $4.806\times10^{-3}$ | 1.000 | 1.000 |
| positive regulation of anoikis | GO:2000210 | $4.806\times10^{-3}$ | 1.000 | 1.000 |
| negative regulation of gene expression, epigenetic | GO:0045814 | $5.753\times10^{-1}$ | 1.000 | $9.109\times10^{-3}$ |
| maintenance of chromatin silencing | GO:0006344 | $2.159\times10^{-2}$ | 1.000 | $5.863\times10^{-1}$ |
| glycerol ether metabolic process | GO:0006662 | $2.527\times10^{-2}$ | 1.000 | 1.000 |
| cilium assembly | GO:0060271 | $6.675\times10^{-2}$ | $2.555\times10^{-2}$ | 1.000 |
| photosystem I assembly | GO:0048564 | $4.400\times10^{-2}$ | 1.000 | 1.000 |
| centrosome cycle | GO:0007098 | $4.851\times10^{-2}$ | $7.005\times10^{-1}$ | $3.609\times10^{-1}$ |

1 to 8 of 8 Page 1 of 1

| ppersica.GO_CC.RABT11E10 | | 200CU x 0CU | 475CU x 0CU | 770CU x 0CU |
|---|---|---|---|---|
| Term Name | Term ID | p_adj | p_adj | p_adj |
| clathrin-coated vesicle | GO:0030136 | $6.173\times10^{-2}$ | $1.670\times10^{-2}$ | $1.757\times10^{-2}$ |
| nuclear microtubule | GO:0005880 | $9.814\times10^{-1}$ | 1.000 | $1.803\times10^{-2}$ |
| cell | GO:0005623 | $1.952\times10^{-2}$ | 1.000 | 1.000 |
| cortical microtubule cytoskeleton | GO:0030981 | $3.616\times10^{-2}$ | 1.000 | 1.000 |

1 to 4 of 4 Page 1 of 1

| ppersica.GO_MF.RABT11E10 | | 200CU x 0CU | 475CU x 0CU | 770CU x 0CU |
|---|---|---|---|---|
| Term Name | Term ID | p_adj | p_adj | p_adj |
| dethiobiotin synthase activity | GO:0004141 | $8.759\times10^{-2}$ | 1.000 | $3.354\times10^{-2}$ |
| AMP deaminase activity | GO:0003876 | $4.229\times10^{-2}$ | 1.000 | 1.000 |

1 to 2 of 2 Page 1 of 1

**CHH**

| ppersica.GO_BP.RABT11E10 | | 200CU x 0CU | 475CU x 0CU | 770CU x 0CU |
|---|---|---|---|---|
| Term Name | Term ID | p_adj | p_adj | p_adj |
| sugar mediated signaling pathway | GO:0010182 | $9.006\times10^{-3}$ | 1.000 | $9.737\times10^{-1}$ |
| plant-type cell wall organization | GO:0009664 | $5.138\times10^{-1}$ | 1.000 | $2.135\times10^{-2}$ |
| regulation of timing of transition from vegetative to reprod... | GO:0048510 | $2.286\times10^{-2}$ | 1.000 | 1.000 |

1 to 3 of 3 Page 1 of 1

| ppersica.GO_CC.RABT11E10 | | 200CU x 0CU | 475CU x 0CU | 770CU x 0CU |
|---|---|---|---|---|
| Term Name | Term ID | p_adj | p_adj | p_adj |
| COPII vesicle coat | GO:0030127 | 1.000 | 1.000 | $2.420\times10^{-3}$ |

1 to 1 of 1 Page 1 of 1

| ppersica.GO_MF.RABT11E10 | | 200CU x 0CU | 475CU x 0CU | 770CU x 0CU |
|---|---|---|---|---|
| Term Name | Term ID | p_adj | p_adj | p_adj |
| primary amine oxidase activity | GO:0008131 | 1.000 | $2.019\times10^{-3}$ | 1.000 |
| lanosterol synthase activity | GO:0000250 | $7.144\times10^{-1}$ | $2.863\times10^{-2}$ | $1.492\times10^{-2}$ |
| beta-amyrin synthase activity | GO:0042300 | $7.144\times10^{-1}$ | $2.863\times10^{-2}$ | $1.492\times10^{-2}$ |
| L-alanine transmembrane transporter activity | GO:0015180 | $3.215\times10^{-2}$ | 1.000 | $9.041\times10^{-1}$ |
| aliphatic-amine oxidase activity | GO:0052595 | 1.000 | $3.807\times10^{-2}$ | 1.000 |
| aminoacetone:oxygen oxidoreductase(deaminating) activity | GO:0052594 | 1.000 | $3.807\times10^{-2}$ | 1.000 |
| phenethylamine:oxygen oxidoreductase (deaminating) activ... | GO:0052596 | 1.000 | $3.807\times10^{-2}$ | 1.000 |
| tryptamine:oxygen oxidoreductase (deaminating) activity | GO:0052593 | 1.000 | $3.807\times10^{-2}$ | 1.000 |

1 to 8 of 8 Page 1 of 1

**Fig. S13** Histograms graphs representing the ABA/GA4 ratio at p≤ 0.05 level at 0 Chilling Units (CU), 200CU, 475CU and 770CU. Error bars indicate standard deviation (+/- SD) and letters indicate differences between time points determined by Tukey's test.

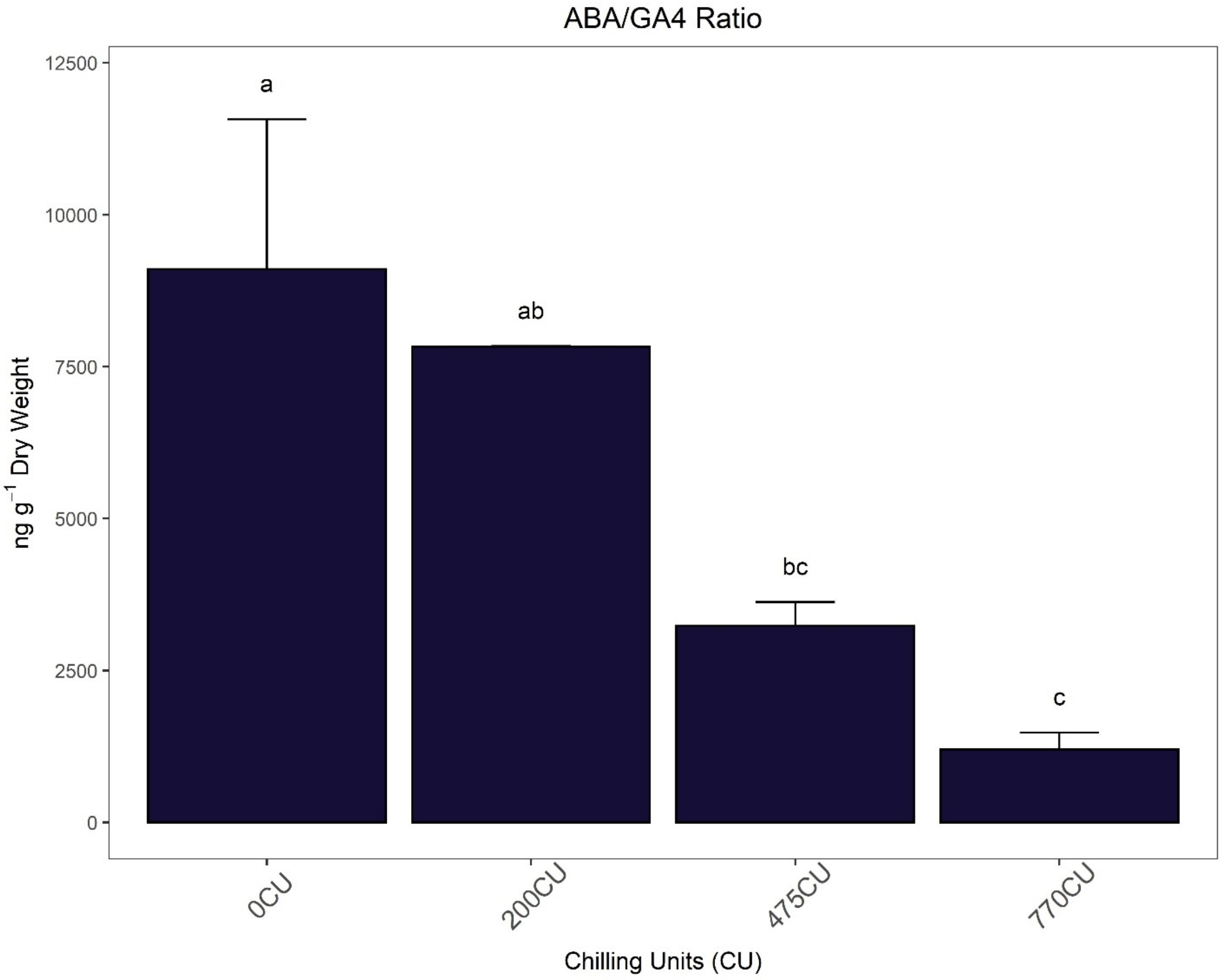

**Fig. S14** Longitudinal sections of peach buds during flower development. Image (a) represent floral buds at 0CU stained with 0.1% Aniline blue. (b) is negative control of aniline signal. Bars = 500µm

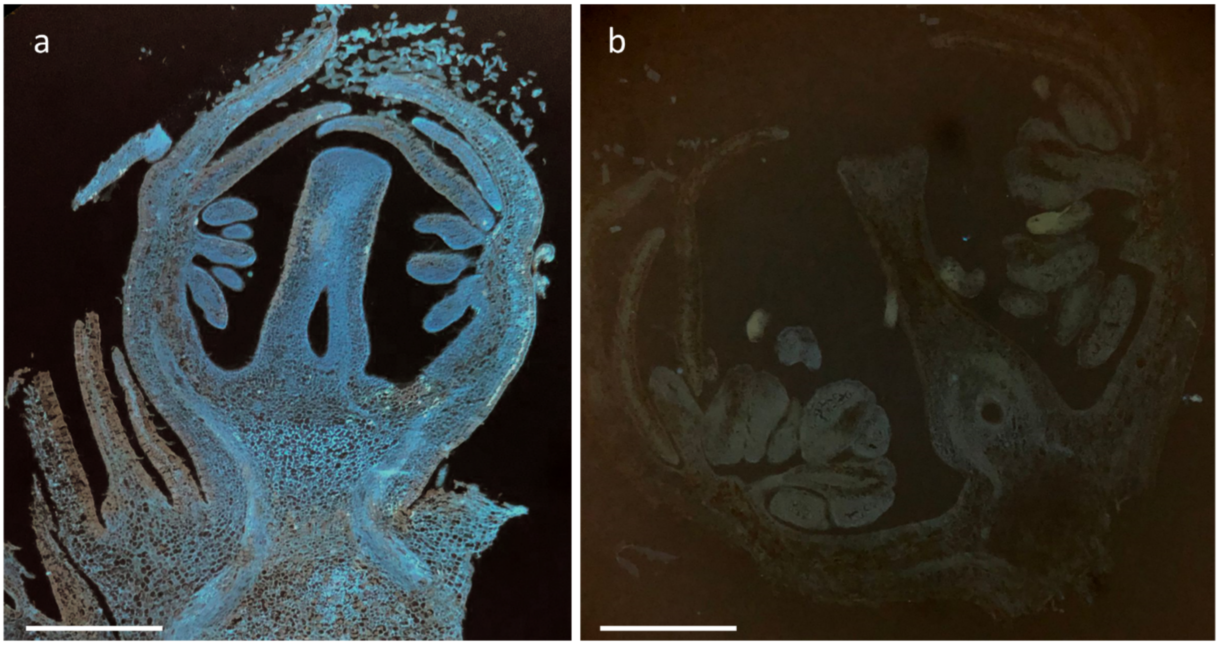

**Table S1** List of primers employed in this work.

| **Table S1** Sequences of primers used in this study | |
|---|---|
| **Gene Name** | **Sequence** |
| PpeDAM6_Forward | 5'- GGTACAAAGCGCACACAAATGATCTCG- 3' |
| PpeDAM6_Reverse | 5'- CAGCTGGTGGAGGTGGCAATTATG- 3' |
| PpeDAM5_Forward | 5'- CCACATCAAACTGAGTAAGGAACTC- 3' |
| PpeDAM5_Reverse | 5'- GCTAACAACCAGCTAAGGCAGACG- 3' |
| PpeDAM4_ Forward | 5'- GAAGAGCTGGATCTGGATGAGTTGC- 3' |
| PpeDAM4_ Reverse | 5'- TCTGATTGTTGGCTTCTACCAGCTCAGT- 3' |
| PpeDAM3_ Forward | 5'- ACCAGCTAAGGCAGACGATGA- 3' |
| PpeDAM3_ Reverse | 5'- GAGGGAGAGAGACTGAGAGCA- 3' |
| PpeGA20ox_Forward | 5'- GCTGAATTACTACCCACCGTGCC- 3' |
| PpeGA20ox_ Reverse | 5'- CAAACACCTCAAGCCCTCCAACT- 3' |
| PpeNCED5_Forward | 5'-ATTTTAGGGTGAGAGGTTTTGGGGG- 3' |
| PpeNCED5_ Reverse | 5'-ATCTCATCTCACGCACCTTTTTGGC- 3' |
| PpeCYP707A4_Forward | 5'-TCACCAAGGAGACTACCACAATAGCCT- 3' |
| PpeCYP707A4_ Reverse | 5'-CAAGGAAGCCAACATCAAAGGAGAACC- 3' |
| PpeDREB1D_Forward | 5'-TAAGGGGGTGGTGAATGAGGAGA- 3' |
| PpeDREB1D_ Reverse | 5'-CAGTTCCCACGTCATTCCAATCCAT- 3' |
| PpeUBQ_ Forward | 5'- AAGGCTAAGATCCAAGACAAAGAG- 3' |
| PpeUBQ_ Reverse | 5'- CCACGAAGACGAAGCACTAAG- 3' |

**Table S2** Prunus GO terms of reference and newly annotated transcripts were de-novo annotated using Trinotate (Bryant *et al*., 2017) and compared with the prunus GO annotation available at EnsemblPlants/Biomart database on July 2020 using WEGO GO plotting tool categorized using level 2 of the GO lineage.

| | | **Reference_Biomart 2020-07-30** | **RABT_ Trinotate** |
|---|---|---|---|
| Gene | | 20.101 | 21.001 |
| Annotated Genes | | 20.101 | 21.001 |
| GO Terms | Biological | 12.019 | 17.668 |
| | Cellular | 11.05 | 17.645 |
| | Function | 15.641 | 18.18 |
| | Total | 38.71 | 53.493 |

**Table S3** List of codes, index adaptors and oligonucleotides.

| | **AciI** | **EcoT22I** | **PstI** | | **Name** | **Oligo sequence** |
|---|---|---|---|---|---|---|
| **1** | FF8_R1_P25 | FF8_R1_P10 | FF8_R1_P1 | index_7 | PCR2_Idx_7_CGATGT | CAAGCAGAAGACGGCATACGAGATGATCTGGTGACTGGAGTTCAGACGTGTGC |
| **2** | FF8_R2_P26 | FF8_R2_P11 | FF8_R2_P2 | | | |
| **3** | FF8_R3_P27 | FF8_R3_P12 | FF8_R3_P3 | | | |
| **4** | FF11_R1_P25 | FF11_R1_P10 | FF11_R1_P1 | index_12 | PCR2_Idx_12_CTTGTA | CAAGCAGAAGACGGCATACGAGATTACAAGGTGACTGGAGTTCAGACGTGTGC |
| **5** | FF11_R2_P26 | FF11_R2_P11 | FF11_R2_P2 | | | |
| **6** | FF11_R3_P27 | FF11_R3_P12 | FF11_R3_P3 | | | |
| **7** | FF13_R1_P25 | FF13_R1_P10 | FF13_R1_P1 | index_14 | PCR2_Idx_14_AGTTCC | CAAGCAGAAGACGGCATACGAGATGGAACTGTGACTGGAGTTCAGACGTGTGC |
| **8** | FF13_R2_P26 | FF13_R2_P11 | FF13_R2_P2 | | | |
| **9** | FF13_R3_P27 | FF13_R3_P12 | FF13_R3_P3 | | | |
| **10** | FF9_R1_P25 | FF9_R1_P10 | FF9_R1_P1 | index_15 | PCR2_Idx_15_ATGTCA | CAAGCAGAAGACGGCATACGAGATTGACATGTGACTGGAGTTCAGACGTGTGC |

| | | | | | | |
|---|---|---|---|---|---|---|
| **11** | FF9_R1_ P26 | FF9_R1_ P11 | FF9_R1 _P2 | | | |
| **12** | FF9_R1_ P27 | FF9_R1_ P12 | FF9_R1 _P3 | | | |

**Table S4** Characteristics of the restriction enzymes used for the MCSeEd technique.

| Enzyme | Recognition site | Cleavage site | Methyl sensitive | Cleavage blocked by | Methyl Context |
|---|---|---|---|---|---|
| AciI | CCGC / GCGG | C'CGC / G'CGG | Yes | C 5mCGC / G 5mCGG | CG |
| PstI | CTGCAG | CTGCA'G | Yes | CTG 5mCAG | CHG |
| EcoT22I | ATGCAT | ATGCA'T | Yes | ATG 5mCAT | CHH |
| MseI | TTAA | T'TAA | No | Not sensitive | - |

**Table S5** Sequencing data summary of DNA methylation sequencing.

| Enzyme | Sample ID | Total sample reads | Unique mapped reads | Useful reads (%) |
|---|---|---|---|---|
| AciI | ff11_a_aci_P25 | 7954662 | 5474988 | 68.83 |
| | ff11_b_aci_P26 | 7241206 | 5151317 | 71.14 |
| | ff11_c_aci_P27 | 7507888 | 5000475 | 66.60 |
| | ff13_a_aci_P25 | 7373074 | 5113836 | 69.36 |
| | ff13_b_aci_P26 | 6185175 | 4286994 | 69.31 |
| | ff13_c_aci_P27 | 8509766 | 5903144 | 69.37 |
| | ff8_a_aci_P25 | 6730952 | 4387580 | 65.19 |
| | ff8_b_aci_P26 | 5345257 | 3535845 | 66.15 |
| | ff8_c_aci_P27 | 6077477 | 3909964 | 64.34 |
| | ff9_a_aci_P25 | 7202216 | 4914323 | 68.23 |
| | ff9_b_aci_P26 | 6746382 | 4716060 | 69.91 |
| | ff9_c_aci_P27 | 8680212 | 5809155 | 66.92 |
| | ff11_a_pstI_P1 | 4867693 | 4046797 | 83.14 |
| | ff11_b_pstI_P2 | 3960294 | 3307900 | 83.53 |
| | ff11_c_pstI_P3 | 5531513 | 4584420 | 82.88 |

| | | | | |
|---|---|---|---|---|
| **PstI** | ff13_a_pstI_P1 | 3869289 | 3263086 | 84.33 |
| | ff13_b_pstI_P2 | 3539286 | 2958903 | 83.60 |
| | ff13_c_pstI_P3 | 4581077 | 3889422 | 84.90 |
| | ff8_a_pstI_P1 | 6772054 | 5404083 | 79.80 |
| | ff8_b_pstI_P2 | 3934093 | 3098230 | 78.75 |
| | ff8_c_pstI_P3 | 4708474 | 3797767 | 80.66 |
| | ff9_a_pstI_P1 | 4825129 | 4013298 | 83.17 |
| | ff9_b_pstI_P2 | 4408025 | 3653957 | 82.89 |
| | ff9_c_pstI_P3 | 4482585 | 3741011 | 83.46 |
| **EcoT22I** | ff11_a_eco_P10 | 4589178 | 2770709 | 60.37 |
| | ff11_b_eco_P11 | 3291022 | 1968646 | 59.82 |
| | ff11_c_eco_P12 | 5738414 | 3482576 | 60.69 |
| | ff13_a_eco_P10 | 4903715 | 2743805 | 55.95 |
| | ff13_b_eco_P11 | 3697103 | 2003721 | 54.20 |
| | ff13_c_eco_P12 | 5913241 | 3373410 | 57.05 |
| | ff8_a_eco_P10 | 4487305 | 2658309 | 59.24 |
| | ff8_b_eco_P11 | 3078440 | 1763084 | 57.27 |
| | ff8_c_eco_P12 | 3568308 | 2174719 | 60.95 |
| | ff9_a_eco_P10 | 3719620 | 2220525 | 59.70 |
| | ff9_b_eco_P11 | 3296702 | 1932096 | 58.61 |
| | ff9_c_eco_P12 | 2253555 | 1381590 | 61.31 |

**Table S6** Means and standard deviation of hormones quantification.

| Chilling Units | GA4 | GA1 | ABA | IAA | DHZ | iP | tZ |
|---|---|---|---|---|---|---|---|
| **0CU** | 0.15 | 0.62 | 1104.6 | 12.87 | 0.18 | 0.008 | 0.042 |
| **0CU** | 0.16 | 0.45 | 1735.8 | 10.98 | 0.086 | 0.013 | 0.032 |
| **0CU** | 0.15 | 0.53 | 355.8 | 9.2 | 0.115 | 0.004 | 0.043 |
| **Mean** | 0.153333 | 0.533333 | 1065.4 | 11.01667 | 0.127 | 0.008333 | 0.039 |
| **Standard Dev.** | 0.005774 | 0.085049 | 690.8346 | 1.835275 | 0.048135 | 0.004509 | 0.006083 |
| | | | | | | | |
| **200CU** | 0.13 | 0.6 | 1157.5 | 11.99 | 0.116 | 0.004 | 0.096 |
| **200CU** | 0.18 | 0.65 | 1410 | 12.1 | 0.1 | 0.005 | 0.033 |
| **200CU** | 0.09 | 0.54 | 704.2 | 9.92 | 0.092 | 0.036 | 0.043 |
| **Mean** | 0.133333 | 0.596667 | 1090.567 | 11.33667 | 0.102667 | 0.015 | 0.057333 |
| **Standard Dev.** | 0.045092 | 0.055076 | 357.6289 | 1.228102 | 0.01222 | 0.018193 | 0.033858 |
| | | | | | | | |
| **475CU** | 0.17 | 0.54 | 596.6 | 16.38 | 0.132 | 0.007 | 0.052 |
| **475CU** | 0.2 | 0.55 | 214.9 | 14.41 | 0.143 | 0.003 | 0.052 |
| **475CU** | 0.21 | 0.67 | 621.3 | 10.93 | 0.1 | 0.042 | 0.043 |
| **Mean** | 0.193333 | 0.586667 | 477.6 | 13.90667 | 0.125 | 0.017333 | 0.049 |
| **Standard Dev.** | 0.020817 | 0.072342 | 227.8398 | 2.759644 | 0.022338 | 0.021455 | 0.005196 |
| | | | | | | | |
| **770CU** | 0.16 | 0.52 | 223.2 | 17.77 | 0.168 | 0.007 | 0.057 |
| **770CU** | 0.22 | 0.58 | 220.4 | 14.94 | 0.168 | 0.058 | 0.035 |
| **770CU** | 0.17 | 0.78 | 502.8 | 11.13 | 0.156 | 0.047 | 0.046 |
| **Mean** | 0.183333 | 0.626667 | 315.4667 | 14.61333 | 0.164 | 0.037333 | 0.046 |
| **Standard Dev.** | 0.032146 | 0.136137 | 162.2415 | 3.332031 | 0.006928 | 0.026839 | 0.011 |

**Table S7** RNA-Seq summary statistics.

| Sample | Raw Reads | Filter rRNA | Filter Viroid | Trimmomatic | Mapped reads | Assigned Reads |
|---|---|---|---|---|---|---|
| **0CU_R1** | 35137978 | 34810465 | 33153296 | 31705652 | 23615797 | 22427388 |
| **0CU_R2** | 30607014 | 29975719 | 28796787 | 27461165 | 20482604 | 18823160 |
| **200CU_R1** | 35398354 | 35241638 | 34231915 | 33070401 | 24534230 | 23162635 |
| **200CU_R2** | 38936842 | 37908325 | 36963825 | 35435382 | 27126737 | 25423199 |
| **475CU_R1** | 35558527 | 34654478 | 33493265 | 32121978 | 24497568 | 22988354 |
| **475CU_R2** | 32985237 | 32516190 | 31400257 | 30097235 | 22344678 | 20883271 |
| **770CU_R1** | 37207522 | 36050286 | 34888822 | 33539838 | 25793818 | 24321151 |
| **770CU_R2** | 31035155 | 30260528 | 29433691 | 28163737 | 21546440 | 20425390 |

New Phytologist

**Table S8** Genes belonging to ABCDE model.

| | | FPKM Values | | | | | | |
|---|---|---|---|---|---|---|---|---|
| **Gene ID AT** | **Gene ID Ppe** | **0CU** | **200CU** | **475CU** | **770CU** | **AT Gene_name** | **Cluster** | **Class** |
| AT1G69120 | PRUPE_3G249300 | 1.114243 | 0.408766 | -0.29696 | -1.22604 | AP1 | 2 | A |
| AT4G36920 | PRUPE_6G231700 | 0.241227 | 1.038683 | 0.082492 | -1.3624 | AP2 | 2 | A |
| AT3G54340 | PRUPE_1G371300 | -1.43871 | 0.140035 | 0.465982 | 0.83269 | AP3 | 1 | B |
| AT5G20240 | PRUPE_1G489400 | -0.89018 | -0.81231 | 0.629327 | 1.073163 | PI | 1 | B |
| AT3G54340 | PRUPE_7G164100 | -1.19168 | -0.45973 | 0.775973 | 0.875443 | AP3 | 1 | B |
| AT4G18960 | PRUPE_4G070500 | -1.1175 | -0.49667 | 0.489497 | 1.124676 | AG | 1 | C |
| AT3G02310 | PRUPE_3G249400 | -1.14836 | -0.45915 | 0.505075 | 1.102433 | SEP2 | 1 | E |
| AT2G45650 | PRUPE_2G151000 | -1.31741 | -0.08032 | 0.323927 | 1.073801 | AGL6 | 1 | E |

**Methods S1**

***in situ* Hybridization**

*In situ* hybridization experiment was performed to localize the *DAM4* expression domains and was conducted as previously described by (Varotto *et al.*, 2003).

Slides were deparaffinized and treated with 10 μ g mL−1 proteinase K. Transcript amplification of DAM4 was performed using the primers present in the Table S1 and designed on coding DNA sequence (CDS). Then, probes were cloned using TOPO® Cloning (Thermo Fisher). In vitro transcription of the DIG-UTP (Roche) labeled RNA sense and antisense probes was obtained using T7 and SP6 polymerases. The hybridization was performed in a 50% formamide buffer at 48˚C overnight. Digoxigenin (DIG) detection and signal visualization were done using Anti-Digoxigenin-AP antibody (Roche) and NBT plus BCIP (Roche), following the manufacturer's instructions. Slides were air-dried and mounted with DPX mounting medium (Fluka Biochemika).

**Hormone Quantification**

Frozen flower buds (200 mg) were grinded and suspended in 80% methanol-1% acetic acid containing internal standards and mixed by shaking during one hour at 4ºC. The extract was kept a -20ºC overnight and then centrifuged and the supernatant dried in a vacuum evaporator. The dry residue was dissolved in 1% acetic acid and passed through a reverse phase column (HLB Oasis 30 mg, Waters), as described in (Seo *et al.*, 2011). For CKs, the extracts were additionally passed through an Oasis MCX (cationic exchange) and eluted with 60% methanol- 5% NH4OH to obtain the basic fraction containing cytokinins. To recover the acid fraction, the MCX cartridge was eluted with methanol. The final residues were dried and dissolved in 5% acetonitrile-1% acetic acid and the hormones were separated by UHPLC with a reverse Accucore C18 column (2.6 μm, 100 mm length; Thermo Fisher Scientific) with an acetonitrile gradient containing 0.05% acetic acid, at 400 μL/min. For GAs and ABA, the gradient was 2 to 55% acetonitrile over 21 min. The hormones were analyzed with a Q-Exactive mass spectrometer (Orbitrap detector; ThermoFisher Scientific) by targeted Selected Ion Monitoring (tSIM; capillary temperature 300ºC, S-lens RF level 70, resolution 70.000) and electrospray ionization (spray voltage 3.0 kV, heater temperature 150ºC, sheath gas flow rate 40 μL/min, auxiliary gas flow rate 10 μL/min) in negative mode for acidic hormones or positive mode for CKs. The concentrations of hormones in the extracts were

determined using embedded calibration curves and the Xcalibur 4.0 and TraceFinder 4.1 SP1 programs. The internal standards for quantification of each of the different plant hormones were the deuterium-labelled hormones, (purchased from OlChemim Ltd, Olomouc, Czech Republic).

**RNA Sequencing (RNA-Seq) and differentially expressed genes (DEG) identification**

DNase digestion using the RNAse-Free DNase Set (Qiagen) was included during RNA isolation. RNA concentration and quality were determined by measuring OD260/230 and OD260/280 ratio on a NanoDrop 2000c spectrophotometer (Thermo Scientific).

For each sample x replicate combination, 30 M paired-end reads of 150 nucleotides were generated. The quality of reads was assessed using FastQC (https://www.bioinformatics.babraham.ac.uk/projects/fastqc/). Initial read quality assessment revealed the peach RNA-Seq libraries contained residual contaminations of rRNA and RNA virus from Peach latent mosaic viroid. The sequenced reads were pre-processed for rRNA and viroid contaminant reads filtering with ERNEFILTER 2.1.1 (Del Fabbro *et al.*, 2013), and then Trimmomatic (Bolger *et al.*, 2014) was applied for adapter clipping and low quality sequence filter and trimming. High quality reads were finally mapped to the *P. persica* genome v.2.0 (Verde *et al.*, 2017) obtained from the Ensembl (http://plants.ensembl.org/index.html; release 43) using the spliced aligner HISAT2 (Kim *et al.*, 2015). Mapped reads were used for Reference Annotation Based Transcript (RABT) assembly of each individual RNA-Seq sample using Stringtie v2.0.4 (Pertea *et al.*, 2015; Kovaka *et al.*, 2019). Reassembled transcriptomes were merged using Stringtie v2.0.4 and then compared and integrated into the reference *Prunus persica* transcriptome annotation using Gffcompare (Pertea & Pertea, 2020) and through a customized Perl script. Final gene annotation allowed the correction of *PpDAM* loci annotation (Supplementary Figure S2) and the identification of 2,846 new bud-expressed loci not included in the reference transcriptome.

Gene expression counts were generate for reference and newly annotated genes using featureCounts software program (Liao *et al.*, 2014) and principal component analysis (PCA) was first used to assessing the biological replicates quality.

The differential expression analysis was carried out using DESeq2 (Love *et al.*, 2014): after estimation of size factors and dispersion between samples and genes, differentially expressed genes were identified applying the likelihood ratio test (LRT). Differently to the default Wald test,

LRT is used to identify any genes that show change in expression across the different levels (CU accumulation), resulting particularly useful in analyzing time course experiments. Genes with a LRT adjusted p-value $\leq 0.01$, and showing a fold change in expression of at least 1.5 (up or down) in the comparison of each stage with the remaining three, were considered as significantly differentially expressed genes (DEGs). Gene clusters exhibiting particular patterns across samples were identified and plotted using the DEGreport R package (Pantano, 2020) using variance stabilizing transformation (VST) expression values as input.
Gene Ontology (GO) enrichment was determined by comparing the number of DEGs included in each cluster to the number of expressed genes in each GO term with gProfiler web-software (Raudvere *et al.*, 2019): the hypergeometric statistic for every term was used to estimate the significance of enriched pathways and processes in the gene lists and the default ontology-focused g:SCS correction method for multiple testing was applied. Prunus GO terms of reference and newly annotated transcripts were *de-novo* annotated using Trinotate (Bryant *et al.*, 2017) and compared with the *Prunus persica* GO annotation available at EnsemblPlants/Biomart database on July 2020 (Supplementary Figure S3 and table S2) using WEGO GO plotting tool (Ye *et al.*, 2018), categorized using level 2 of the GO lineage.

**RNA-Seq Validation**

Total RNA was extracted following the previously cited protocol. cDNA synthesis was performed with the SuperScript III reverse transcriptase kit (Invitrogen) according to the manufacturer's instructions. Quantitative Real-Time PCR expression analysis was performed using a StepOnePlus™ Real-Time PCR System (Applied Biosystems) and the FAST SYBR® GREEN PCR Master Mix (Thermo Fisher Scientific), following the manufacturer's guidelines. Melting curves analysis revealed a single amplification product in each reaction. Three technical replicates were carried out for each primer combination in each sample and an absolute quantification of gene expression (normalized to UBIQUITIN –UBQ- transcript quantities) was performed with the StepOne Software 2.3 (Thermo Fisher Scientific). Primer sequences, specifically designed on each target gene (*PpeDAM6*, *PpeDAM5*, *PpeDAM4*, *PpeDAM3*, *PpeDREB1D*, *PpeCYP707A4, PpeNCED5, PpeGA20ox*, and *PpeUBQ*), are reported in Table S1.

**Chromatin Immunoprecipitation Sequencing (ChIP-Seq) analysis**

FastQC (https://www.bioinformatics.babraham.ac.uk/projects/fastqc/) was used to assess the quality of the reads. The ChIP-Seq raw reads were processed for adapter clipping and quality score trimming using Trimmomatic v 0.39 (Bolger *et al.*, 2014). Clean reads were mapped to the *P. persica* genome v.2.0 (Verde *et al.*, 2017) obtained from Ensembl (http://plants.ensembl.org/index.html) with bowtie2 v2.4.1 (Langmead & Salzberg, 2012); soft-trimming (5 bp at 5' and 10 bp at 3') was enabled. Reads with MAPQ > 10 were used for the subsequent analysis. Aligned reads were sorted using SAMtools v.1.3 and duplicated reads were removed using Picard v.2.16.0 (http:// broadinstitute.github.io/picard/). ChIP-Seq peak calling and differential binding analysis were performed using Model-based Analysis of ChIP-Seq (MACS2) (Zhang *et al.*, 2008; Feng *et al.*, 2012). Uniquely mapped and not duplicated reads were used for peaks calling with the "*callpeak*" subcommand for each immunoprecipitated sample/replicate with respect to the input control, replicate signals were combined with the "*cmbreps*" subcommand using the Fisher's combined probability test prior to differential peak enrichment analysis using the "bdgdiff" tool. Identified peaks and differentially enriched peaks were associated with nearby genes using HOMER v4.11 software (Heinz *et al.*, 2010). Correlation heatmaps between replicates, using read count data were produced using DiffBind R package version 4.2 (Fig. S4; Stark & Brown, 2011; Ross-Innes *et al.*, 2012). Library corresponding to replicate R2 of H3K4me3 was not included into analysis pipeline due to it low quality.

**Library preparation and sequencing for DNA methylation analysis**

On-Column RNase Digestion was performed. DNA concentration and quality were determined by measuring OD260/230 and OD260/280 ratio, respectively, on a NanoDrop 2000c spectrophotometer (Thermo Scientific) and using Qubit 2.0 Fluorometer (Invitrogen, Carlsbad, CA). Illumina sequencing was performed at Novogene (HK) Company Limited according to the standard operation. Since a methylation-sensitive enzyme cannot digest methylated site, the read count at a specific locus is expected to anticorrelate to genomic methylation level, this is efficiently used for estimating differential methylation changes over different genomic regions between two samples (Marconi *et al.*, 2019). The raw reads were checked by quality analysis using the FastQC (www.bioinformatics.babraham.aC.uk/projects/fastqc/, accessed on 30 May 2020) program and ambiguous and poor-quality reads (with a base count of Phred value <20), were removed using the

TrimGalore program (https://www.bioinformatics. babraham.ac.uk/projects/trim_galore, accessed on 30 May 2020).

## References

**Bolger AM, Lohse M, Usadel B**. **2014**. Trimmomatic: A flexible trimmer for Illumina sequence data. *Bioinformatics* **30**: 2114–2120.
**Bryant DM, Johnson K, DiTommaso T, Tickle T, Couger MB, Payzin-Dogru D, Lee TJ, Leigh ND, Kuo TH, Davis FG, *et al.*** **2017**. A Tissue-Mapped Axolotl De Novo Transcriptome Enables Identification of Limb Regeneration Factors. *Cell Reports* **18**: 762–776.
**Del Fabbro C, Scalabrin S, Morgante M, Giorgi FM**. **2013**. An extensive evaluation of read trimming effects on illumina NGS data analysis. *PLoS ONE* **8**: e85024.
**Feng J, Liu T, Qin B, Zhang Y, Liu XS**. **2012**. Identifying ChIP-seq enrichment using MACS. *Nature Protocols* **7**: 1728–1740.
**Heinz S, Benner C, Spann N, Bertolino E, Lin YC, Laslo P, Cheng JX, Murre C, Singh H, Glass CK**. **2010**. Simple Combinations of Lineage-Determining Transcription Factors Prime cis-Regulatory Elements Required for Macrophage and B Cell Identities. *Molecular Cell* **38**: 576–589.
**Kim D, Langmead B, Salzberg SL**. **2015**. HISAT: A fast spliced aligner with low memory requirements. *Nature Methods* **12**: 357–360.
**Kovaka S, Zimin A V., Pertea GM, Razaghi R, Salzberg SL, Pertea M**. **2019**. Transcriptome assembly from long-read RNA-seq alignments with StringTie2. *Genome Biology* **20**: 1–13.
**Langmead B, Salzberg SL**. **2012**. Fast gapped-read alignment with Bowtie 2. *Nature Methods* **9**: 357–359.
**Liao Y, Smyth GK, Shi W**. **2014**. FeatureCounts: An efficient general purpose program for assigning sequence reads to genomic features. *Bioinformatics* **30**: 923–930.
**Love MI, Huber W, Anders S**. **2014**. Moderated estimation of fold change and dispersion for RNA-seq data with DESeq2. *Genome Biology* **15**: 550.
**Marconi G, Capomaccio S, Comino C, Acquadro A, Portis E, Porceddu A, Albertini E**. **2019**. Methylation content sensitive enzyme ddRAD (MCSeEd): a reference-free, whole genome profiling system to address cytosine/adenine methylation changes. *Scientific Reports* **9**: 1–12.
**Pantano L**. **2020**. *DEGreport: Report of DEG analysis. R package version 1.27.0*, http://lpantano.github.io/DEGreport/.
**Pertea G, Pertea M**. **2020**. GFF Utilities: GffRead and GffCompare. *F1000Research* **9**: 304.
**Pertea M, Pertea GM, Antonescu CM, Chang TC, Mendell JT, Salzberg SL**. **2015**. StringTie enables improved reconstruction of a transcriptome from RNA-seq reads. *Nature Biotechnology* **33**: 290–295.
**Raudvere U, Kolberg L, Kuzmin I, Arak T, Adler P, Peterson H, Vilo J**. **2019**. G:Profiler: A web server for functional enrichment analysis and conversions of gene lists (2019 update). *Nucleic Acids Research* **47**: W191–W198.
**Ross-Innes CS, Stark R, Teschendorff AE, Holmes KA, Ali HR, Dunning MJ, Brown GD, Gojis O, Ellis IO, Green AR, *et al.*** **2012**. Differential oestrogen receptor binding is associated with clinical outcome in breast cancer. *Nature* **481**: 389–393.
**Seo M, Jikumaru Y, Kamiya Y**. **2011**. Profiling of hormones and related metabolites in seed dormancy and germination studies. *Methods in Molecular Biology* **773**: 99–111.

**Stark R, Brown G**. **2011**. DiffBind : differential binding analysis of ChIP-Seq peak data.
**Varotto S, Locatelli S, Canova S, Pipal A, Motto M, Rossi V**. **2003**. Expression Profile and Cellular Localization of Maize Rpd3-Type Histone Deacetylases during Plant Development. *Plant Physiology* **133**: 606–617.
**Verde I, Jenkins J, Dondini L, Micali S, Pagliarani G, Vendramin E, Paris R, Aramini V, Gazza L, Rossini L, *et al.* 2017**. The Peach v2.0 release: High-resolution linkage mapping and deep resequencing improve chromosome-scale assembly and contiguity. *BMC Genomics* **18**: 225.
**Ye J, Zhang Y, Cui H, Liu J, Wu Y, Cheng Y, Xu H, Huang X, Li S, Zhou A, *et al.* 2018**. WEGO 2.0: A web tool for analyzing and plotting GO annotations, 2018 update. *Nucleic Acids Research* **46**: W71–W75.
**Zhang Y, Liu T, Meyer CA, Eeckhoute J, Johnson DS, Bernstein BE, Nussbaum C, Myers RM, Brown M, Li W, *et al.* 2008**. Model-based analysis of ChIP-Seq (MACS). *Genome Biology* **9**: 1–9.
